# Ancestral Karyotype Reconstruction Reveals Chromosome Rearrangements and Large Inversions in *Oryza*

**DOI:** 10.3390/plants15192989

**Published:** 2026-09-30

**Authors:** Qiang Xu, Meifang Lan, Ying Chen, Hongshuang Jiang, Yifan Yu, Aiping Wang, Weihong Wu, Qi Dong

**Affiliations:** 1 College of Agriculture, Shanxi Agricultural University, Taigu 030801, China; 2 Shenzhen Branch, Guangdong Laboratory of Lingnan Modern Agriculture, Genome Analysis Laboratory of the Ministry of Agriculture and Rural Affairs, Agricultural Genomics Institute at Shenzhen, Chinese Academy of Agricultural Sciences, Shenzhen 518120, China; 3 College of Plant Science & Technology, Huazhong Agricultural University, Wuhan 430070, China; 4 State Key Laboratory of Plant Diversity and Specialty Crops and Key Laboratory of Systematic and Evolutionary Botany, Institute of Botany, The Chinese Academy of Sciences, Beijing 100093, China; 5 College of Life Sciences, University of Chinese Academy of Sciences, Beijing 100049, China; 6 China National Botanical Garden, Beijing 100093, China

**Keywords:** *Oryza*, ancestral karyotype reconstruction, chromosome evolution, structural variation, large inversion, comparative genomics

## Abstract

A stable chromosome count does not imply a static genome. *Oryza* species have largely retained a basic chromosome number of 12, yet most rearrangements are described against extant references, making ancestral and derived orientations difficult to distinguish. We reconstructed an ancestral *Oryza* karyotype (AOK) containing 12 chromosomes and 23,010 ordered genes from 24 chromosome-level genome assemblies representing 34 ingroup genome/subgenome units. Unlike a modern reference, the AOK provides gene-order polarity, allowing rearrangements detected in different species and population panels to be compared in the same evolutionary direction. AOK-polarized analysis of 400 rice assemblies revealed structural change at two scales. Across species, the derived allotetraploid karyotypes of *Oryza alta* and *Oryza grandiglumis* were parsimoniously accounted for by three reciprocal translocations. Within rice populations, four principal inversion-associated loci showed contrasting lineage distributions. The most complex locus, on chromosome 6, comprised nested structural haplotypes that were unevenly distributed among rice populations and associated with published variation in seedling cold tolerance. These findings show how lineage-specific chromosome exchanges and polymorphic inversion complexes have reshaped a numerically conserved karyotype. More broadly, an ancestral gene-order reference links deep karyotype evolution with standing structural diversity and provides a framework for tracing the direction of chromosome rearrangements during rice diversification.

## 1. Introduction

The rho (ρ) whole-genome duplication in grasses occurred approximately 90 million years ago in a lineage with seven ancestral chromosomes [1]. Diploidization and subsequent rearrangement produced a 12-chromosome post-rho karyotype [1,2,3]. Broad chromosome correspondence between *Oryza* and *Leersia* suggests that Oryzinae inherited this organization [1].

Paralogous relationships left by rho remain recognizable between several rice chromosomes, including Chr1/Chr5, Chr8/Chr9 and Chr11/Chr12 [1]. The homoeologous regions of Chr11 and Chr12 were initially interpreted as a recent segmental duplication. Comparative evidence instead supports recurrent long-range ectopic recombination and megabase-scale gene conversion across *Oryza* [4]. The proposed initiation region for recurrent gene conversion lies approximately 2.1 Mb from the distal ends of the Chr11 and Chr12 short arms and is extensively rearranged. Such exchanges can produce alignment patterns that resemble structural rearrangements, making breakpoint evidence essential for their classification.

*Oryza* includes diploid and polyploid lineages spanning approximately 15 million years of divergence, yet its diploid species generally retain a haploid chromosome number of 12 [5]. Although chromosome number has remained stable, chromosome structure has changed through lineage-specific rearrangements and exchanges between subgenomes. Much of the gene order nevertheless remains conserved [6,7].

A variation map of 10,548 rice accessions cataloged 54,378,986 single-nucleotide polymorphisms and 11,119,947 insertion/deletion variants, approximately 65.5 million small variants in total, together with 184,736 presence–absence variants, representing sequences present in some accessions but absent in others [8]. Rare alleles accounted for most of this diversity. Long-read panels have resolved gene gain and loss, large inversions and other presence–absence variation [9,10,11]. Pan-TE maps catalog transposable element variation across accessions, whereas pan-tandem-repeat maps catalog variation in tandemly repeated sequences. Both connected repeat variation with gene expression and agronomic traits [12,13]. A centromere map documented chromosome- and subpopulation-level differences in satellite and retrotransposon composition [14].

Large inversions can suppress recombination between alternative arrangements and maintain linked alleles across extended haplotypes [15,16]. In plants, they have been associated with local adaptation, coordinated regulatory effects and ecotypic differentiation [17,18,19]. Rearrangements also contribute to supergene architectures in other systems [20]. Rice pangenome studies have linked inversions to recombination, linkage disequilibrium and gene-expression differences [21,22]. Other population analyses connected large structural variants with Geng/*japonica* differentiation and breeding history [23].

Conserved orthology and synteny can recover ancestral chromosome composition and gene order [2,24,25,26]. These reconstructions have clarified allopolyploid histories, chromosome fusions and lineage relationships in Malvaceae and Brassicaceae, and have resolved the order of chromosome rearrangements in Triticeae [24,25,27]. Unlike an extant reference, an ancestral gene order provides explicit polarity: it distinguishes ancestral-like from derived arrangements and permits comparisons without privileging the state of a single living genome. In rice, however, large structural variants have been reported repeatedly, in domestication, reference-genome and pangenome studies and in large multi-parent populations [9,10,11,21,22,23,28,29], but the descriptions are not always mutually consistent: the orientation, boundaries and labels assigned to a given region depend on the reference used, and the direction and relative sequence of change therefore remain difficult to establish. This study addresses that gap by using reconstructed ancestral gene order to interpret both lineage-specific rearrangements and population structural variation.

The structurally diverse pericentromeric region of chromosome 6 (Chr6) illustrates this problem. Broad and local orientation differences have been reported in reference-genome, domestication and pangenome comparisons [9,21,23,28]. Definitions based on different extant references can reverse the apparent orientation or assign different labels to related configurations. An ancestral gene-order reference separates physical position from evolutionary polarity. It can therefore place overlapping configurations on a common directional axis, allowing nested states and alternative breakpoint topologies to be compared as parts of the same structural series.

We reconstructed an ancestral *Oryza* karyotype (AOK) from 24 chromosome-level genome assemblies representing 34 ingroup genome/subgenome units and linked its gene order to telomere-to-telomere Nipponbare (NIP-T2T) coordinates. We then used the AOK as a directional reference, rather than as a nucleotide surrogate, to polarize chromosome-scale rearrangements across *Oryza* and classify inversion-associated configurations in 400 assemblies. This design allowed genus-scale exchanges and population variants to be compared in a shared evolutionary orientation. At the nested Chr6 hotspot, we compared breakpoint compatibility, local topology and population distribution before examining the major structural haplotypes against published seedling cold-tolerance phenotypes.

## 2. Results

### 2.1. Reconstruction and Genome-Wide Projection of the Oryza Ancestral Karyotype

**Figure 1:**
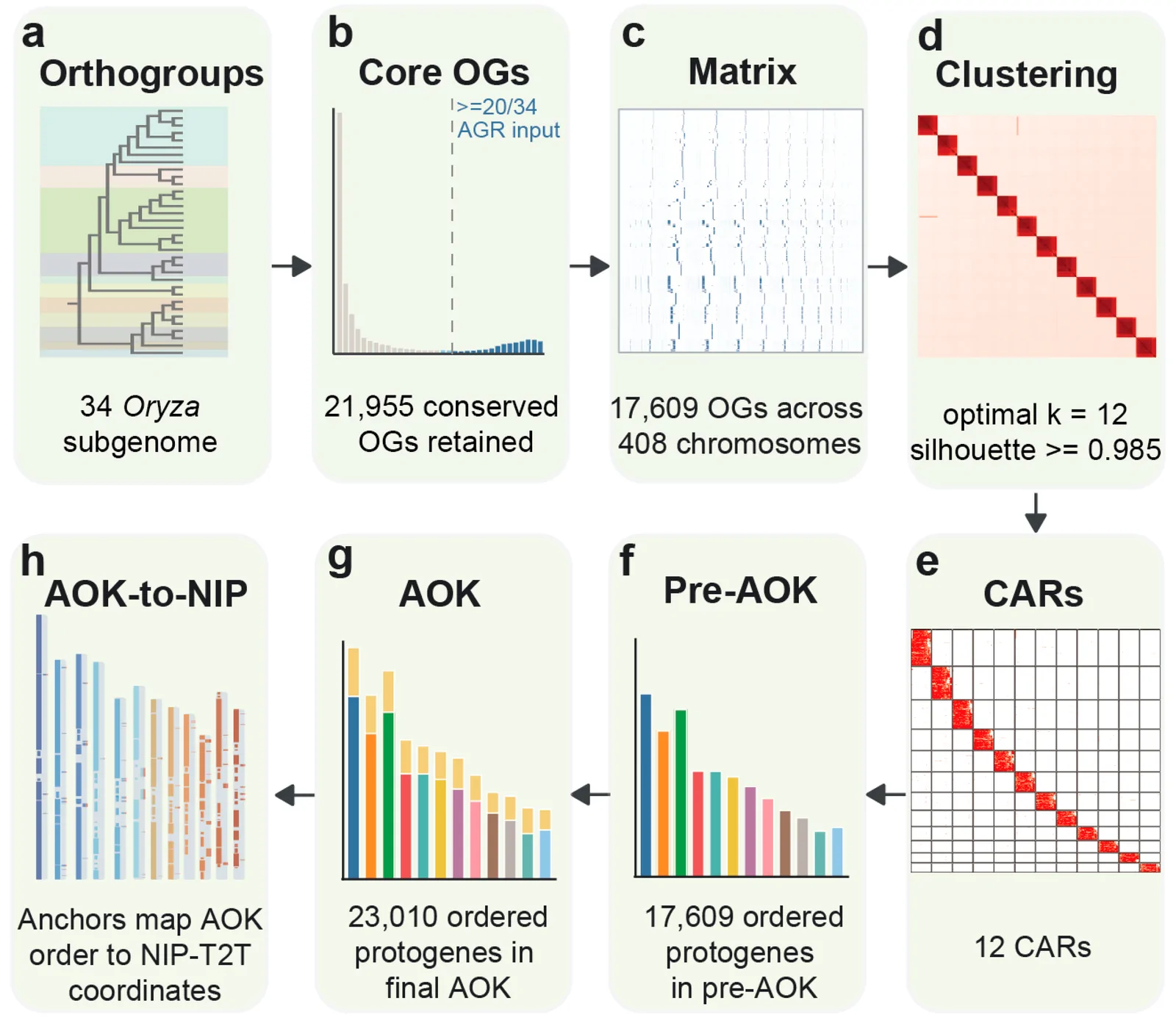
Reconstruction of the ancestral *Oryza* karyotype. (**a**) Orthogroup inference across 34 ingroup genome/subgenome units. (**b**) Filtering of conserved orthogroups. (**c**) Chromosome-by-orthogroup matrix used for AGR clustering. (**d**) Hierarchical clustering of 12 chromosome groups. (**e**) Extraction of 12 contiguous ancestral regions. (**f**) Protogene order in the pre-AOK. (**g**) Final AOK comprising 23,010 ordered protogenes on 12 ancestral chromosomes. (**h**) Orthogroup anchors between AOK gene order and NIP-T2T physical coordinates.

The reconstruction used orthogroup occupancy, chromosome position and conserved synteny across 34 ingroup genome/subgenome units derived from 24 chromosome-level genome assemblies. These data yielded an ancestral *Oryza* karyotype (AOK) of 23,010 ordered protogenes on 12 protochromosomes (Figure 1a–g; Table S1). Conserved orthologous anchors linked protogene rank, used to infer ancestral order and polarity, to the physical coordinates of the corresponding NIP-T2T segments (Figure 1h).

**Figure 2:**
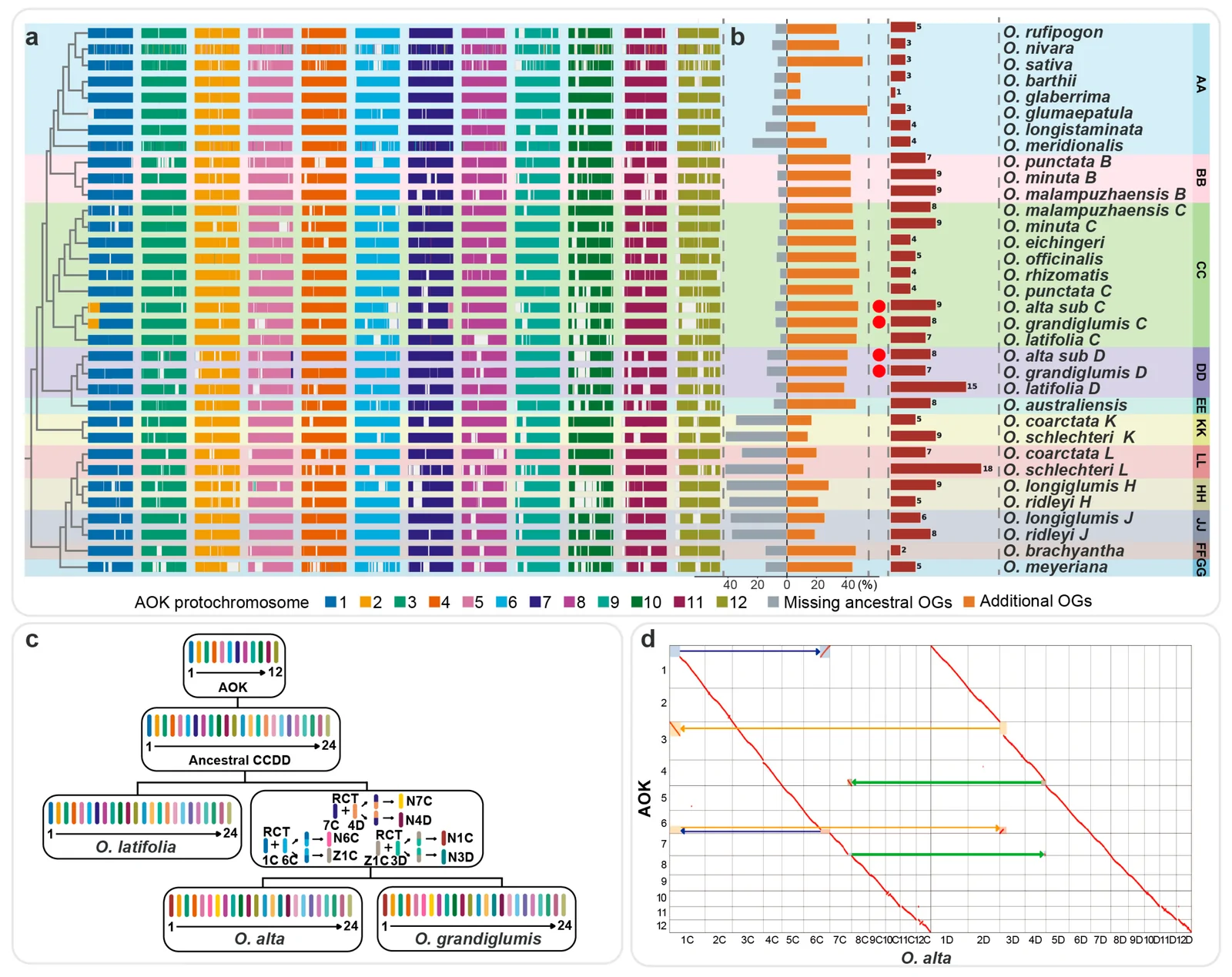
CCDD karyotype remodeling in *Oryza*. (**a**) Chromosome painting of *Oryza* genomes using the 12 AOK chromosomes. (**b**) Missing and additional orthogroups and AOK-polarized reverse-orientation segments for each analytical unit. Gray and orange bars denote missing and additional orthogroups, respectively; red bars count large reverse-orientation segments. Red dots mark interchromosomal structural variation; letters indicate genome types (e.g., AA, BB, CC). (**c**) Diagram of the three-RCT configuration in *Oryza alta* and *Oryza grandiglumis*, with *Oryza latifolia* included for comparison. (**d**) *O. alta*-to-AOK anchor dot plot for the translocated segments.

Most extant chromosomes showed near one-to-one correspondence with the 12 ancestral chromosomes (Figure 2a). Broad chromosome identity was therefore maintained across the 34 ingroup genome/subgenome units despite gene-content turnover and local orientation changes.

Cross-chromosome departures from the AOK were most extensive in CCDD genomes. Reverse-orientation segments also recurred near the homoeologous short arms of Chr11 and Chr12. Because long-range ectopic gene conversion can generate similar alignments, these segments were treated separately rather than counted as ordinary inversions or translocations [4].

### 2.2. Three Reciprocal Translocations in CCDD Karyotype Evolution

Because most cross-chromosome departures were concentrated in the CCDD lineage, we examined these genomes separately. AOK-polarized comparison distinguished conserved chromosome identity from lineage-specific reconfiguration (Figure 2a).

Missing and additional orthogroups were compared for each analytical unit alongside AOK-polarized reverse-orientation segments (Figure 2b). Their distributions did not consistently coincide with chromosome-scale rearrangements, arguing against extensive gene-content divergence as a general feature of these events.

Within the CCDD lineage, *Oryza latifolia* retained ancestral-like chromosome correspondence, whereas *Oryza alta* and *Oryza grandiglumis* shared a derived configuration (Figure 2c). Previous analyses against extant references recovered similar cross-chromosome correspondences but proposed different evolutionary models. Long et al. described a reciprocal translocation between 3D and 1C, together with translocations involving 1C–6C and homoeologous groups 4 and 7 [6]. Fornasiero et al. represented the corresponding pattern as five unbalanced translocations relative to *Oryza sativa* [7]. Explicit AOK polarity integrated these reference-relative signals into a parsimonious model of three reciprocal chromosome translocations (RCTs; Figure 2c). In AOK coordinates, the exchanges involved terminal segments of Chr1C and Chr6C, Chr3D and Chr1C, and Chr4D and Chr7C. These assignments were visible in the representative *O. alta* AOK dot plot (Figure 2d and Figure S1). The distribution of the shared configuration places the three-RCT model after *O. latifolia* diverged and before *O. alta* and *O. grandiglumis* separated.

### 2.3. An AOK-Polarized Scan Identifies Four Principal Inversion-Associated Loci Across 400 Rice Assemblies

**Figure 3:**
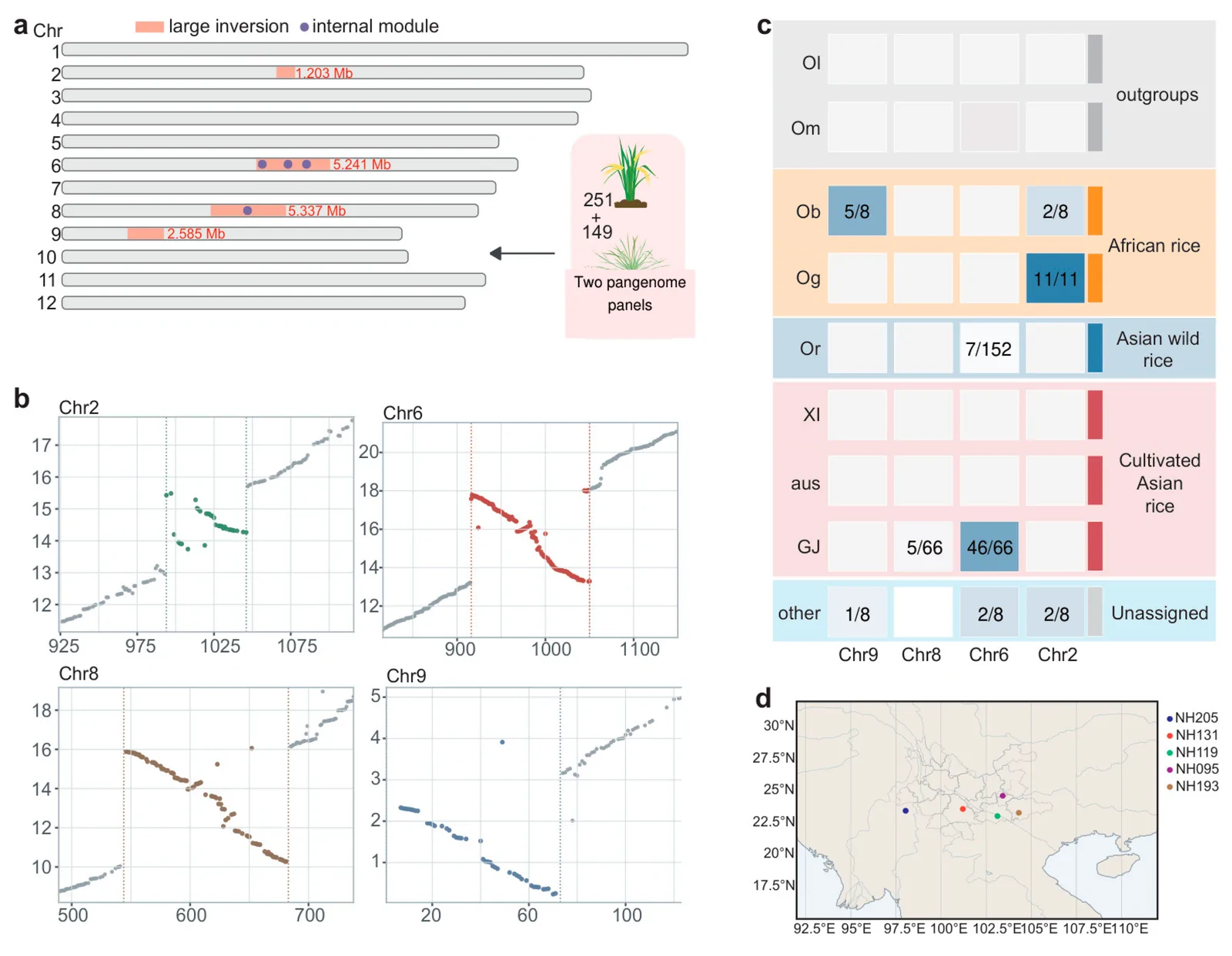
Principal inversion-associated loci in rice assemblies. (**a**) Positions of megabase-scale and local inversion-associated loci across the 12 chromosomes in the 400-assembly panel. (**b**) Representative AOK-polarized dot plots for the retained Chr2, Chr6, Chr8 and Chr9 loci. (**c**) Carrier counts across population groups, with GJ pooling GJ1–GJ5 and GJ-unassigned; only the Big interval is included for Chr6 (darker shading indicates higher carrier frequencies). (**d**) Reported origins of the five Chr8 carriers.

Having resolved lineage-specific translocations at the genus scale, we next asked whether inversion-associated structural variants also segregated within cultivated and wild rice. The combined panel contained 400 assemblies from the 251- and 149-assembly resources; all were retained, with locus frequencies calculated from callable assemblies. The AOK-polarized scan retained four principal inversion-associated loci on Chr2, Chr6, Chr8 and Chr9 (Figure 3a; Table S2).

Representative AOK-polarized dot plots showed intervals of 1.203 Mb on Chr2, 5.241 Mb at the broad Chr6 hotspot, 5.337 Mb on Chr8 and 2.585 Mb on Chr9 (Figure 3b). Event rules and sample-level states are provided in Table S2.

Chr1 and Chr5 signals were not retained as principal configurations. Each compatible event was supported by three or fewer accessions, and these signals were not included in the retained principal-locus state matrix.

**Figure 4:**
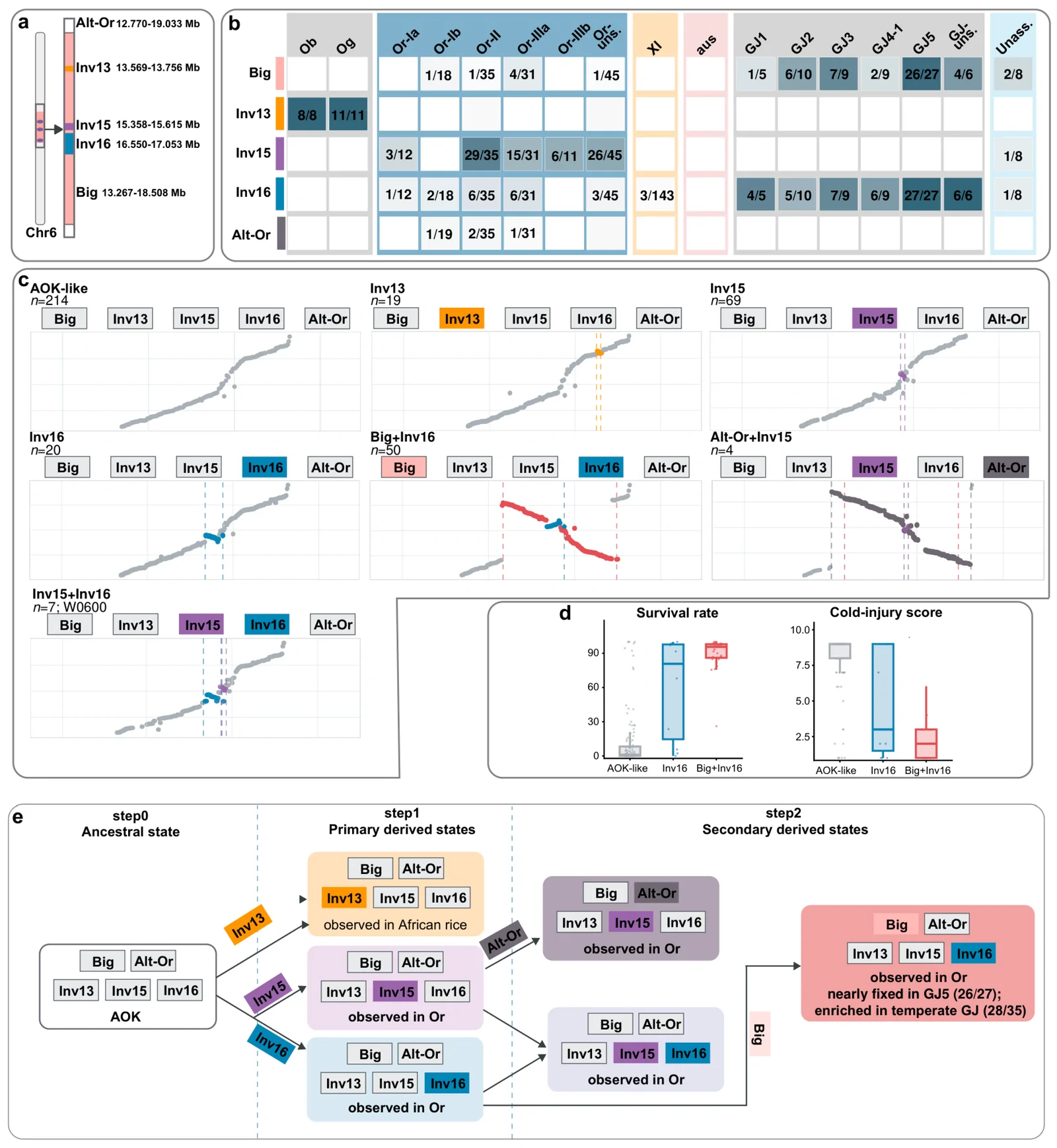
AOK-polarized structural states at the Chr6 hotspot. (**a**) NIP-T2T positions of the Big interval, Inv13, Inv15, Inv16 and the Alt-Or topology. (**b**) Carrier counts across population groups (darker shading indicates higher carrier frequencies). (**c**) Representative AOK-coordinate configurations; W0600 represents Inv15+Inv16. Counts use 395 callable assemblies, except Alt-Or, which was assessed in all 400. (**d**) Seedling survival rate and cold-injury score for AOK-like, Inv16-only and Big+Inv16 structural haplotypes. Points indicate accessions; boxes show medians and interquartile ranges, with whiskers extending to 1.5 times the interquartile range. (**e**) Schematic ordering of the observed structural states; arrows indicate inferred steps rather than dated transitions.

The GJ1–GJ5 labels follow the 10,548-genome subgroup classification, whereas temperate GJ (GJ-tmp) and the other ecotype labels follow the independent 3K K9 classification. Source-matched assignments are reported separately in Table S2a. Six *japonica* assemblies without subgroup assignments were classified as GJ-unassigned, and eight accessions without supported population assignments were retained as unassigned. The four loci had distinct lineage distributions (Figure 3c). The Chr2 locus occurred in all 11 *Oryza glaberrima* assemblies, two of eight *Oryza barthii* assemblies and two of eight unassigned accessions. The Chr9 locus occurred in five of eight *O. barthii* assemblies and one of eight unassigned accessions. Chr8 occurred in five of 60 GJ1–GJ5 assemblies, none of six GJ-unassigned assemblies and none of eight unassigned accessions. For Chr6, Figure 3c summarizes the Big interval, whereas the internal modules Inv13, Inv15 and Inv16 and the breakpoint-distinct Alt-Or topology are resolved in Figure 4.

The five Chr8 carriers (NH095, NH119, NH131, NH193 and NH205) formed a provenance cluster centered on Yunnan Province, China, and nearby regions (Figure 3d). This descriptive pattern was not tested for local adaptation.

Before focusing on Chr6, we asked whether the four principal inversion-associated loci shared functional annotations or repeat-related protein domains. Pfam analysis detected significant enrichment of TE-related domains at both Chr6 and Chr8, with ATHILA, rve, RVT_1 and RVT_3 shared between the loci. No GO or KEGG term reached FDR < 0.05 at either locus, and breakpoint-proximal genes showed no consistent functional enrichment.

### 2.4. AOK-Polarized Chr6 States Resolve Branching Structural Diversity at a Multiallelic Inversion-Complex Locus

Among the four principal inversion-associated loci, Chr6 showed the most complex and phylogenetically structured pattern. Previous descriptions were based on different extant references and population panels [9,21,23,28]. AOK-polarized comparison expressed these configurations relative to the same ancestral orientation, enabling their breakpoint relationships to be compared directly (Figure 4a).

We tracked five structural features at this locus. The Big interval spans 13.267–18.508 Mb in projected NIP-T2T coordinates. The three internal inversion modules—Inv13, Inv15 and Inv16—occupy 13.569–13.756, 15.358–15.615 and 16.550–17.053 Mb, respectively. The breakpoint-distinct Alt-Or topology extends from approximately 12.770 to 19.033 Mb and is defined by two breakpoints outside the Big interval.

Among callable assemblies, canonical Inv13 was detected only in African rice. Six of eight *O. barthii* assemblies and all 11 *O. glaberrima* assemblies carried the full configuration, and two additional *O. barthii* assemblies carried a shared partial subtype (Figure 4b,c). The Inv13 family therefore comprised 19 callable African-rice assemblies. Breakpoint-distinct left-region configurations are listed separately in Figure S2 and Table S2.

The Inv15 module was concentrated in *Oryza rufipogon*-related groups. Among 395 callable assemblies, 80 carried Inv15, including minor boundary subhaplotypes (Figure 4b; Table S2). Seven accessions carried Inv15+Inv16: CW14, CW19, W0600, W1748, W1895, W2197 and W2308. The Alt-Or topology occurred in four *O. rufipogon*-related accessions: one Or-Ib configuration, two Or-II configurations and one Or-IIIa configuration. Topology review placed all four on the Inv15-associated branch, although their outer breakpoints defined Alt-Or as a separate topology (Figure 4b,c and Figure S2). Alt-Or+Inv15 and Inv15+Inv16 therefore represent distinct structural states without canonical Inv13.

The Big interval usually occurred with an Inv16-associated state in GJ. Twenty-six of 27 callable GJ5 assemblies carried the Big interval, and all 27 carried the Inv16-associated state; both were also frequent in GJ1–GJ4 (Figure 4b). Across the 395 callable assemblies, 50 of 55 Big-interval carriers had the Big+Inv16 structural state, although Inv16-associated states also occurred without Big. Big+Inv16 was present in 28 of the 35 temperate GJ (GJ-tmp) accessions in the 251-genome panel (Table S2a). Among the observed structural states, this asymmetry supports a branch in which Inv16 precedes Big (Figure 4e).

Here, structural state refers to an individual configuration, whereas structural haplotype refers to a recurrent combination of the four principal features. Figure 4c summarizes seven recurrent structural states at the Chr6 hotspot. Configurations matching the ancestral AOK order across the entire hotspot were designated AOK-like (*n* = 214). The remaining states were Inv15 without Alt-Or or Inv16 (*n* = 69), Big+Inv16 (*n* = 50), Inv16-only (*n* = 20), the Inv13 family (*n* = 19), Inv15+Inv16 (*n* = 7) and Alt-Or+Inv15 (*n* = 4). AOK-like predominated in indica and several wild-rice groups, whereas Inv13, Inv15 and Big+Inv16 were associated with African rice, *O. rufipogon*-related groups and GJ, respectively. Five Big-only assemblies and other low-frequency or breakpoint-distinct configurations are listed in Table S2.

To place these population-stratified structural states in a phenotypic context, we intersected the Chr6 states with published seedling cold-tolerance records [30]. Records were available for 170 matched accessions with callable Chr6 states. The primary comparison retained 128 AOK-like, 11 Inv16-only and 29 Big+Inv16 accessions. Two Big-only accessions were excluded because the group was too small for a separate estimate. Median survival rate was 0.99%, 80.77% and 95.65% in AOK-like, Inv16-only and Big+Inv16 accessions, respectively (Kruskal–Wallis *p* = 1.6 × 10^−15^). Median cold-injury scores were 9, 3 and 2 in the same order (*p* = 4.6 × 10^−17^; Figure 4d; Table S2b). Both Inv16-bearing groups shifted in the same direction relative to AOK-like accessions. Because these structural haplotypes were strongly stratified by population, the comparison was treated as descriptive rather than as evidence for an effect of either inversion.

## 3. Discussion

### 3.1. An Ancestral Reference for Genus- and Population-Scale Comparisons

Together, these results show that a stable chromosome count does not imply a static genome. In *Oryza*, numerical stability masked lineage-specific translocations and extensive differences in segment orientation, whereas broad chromosome identity was nevertheless maintained during diversification, consistent with previous comparative studies [5,7]. The comparisons above combine three kinds of information that are easily confused with one another. Protogene order in the AOK provides a reference for polarizing segment orientation; the position of that segment in the NIP-T2T assembly gives its physical location; and the population assemblies show how often each structural state occurs. The AOK helps distinguish ancestral and derived arrangements without assuming that the modern reference retains the ancestral state. The distinction matters at both scales examined here.

At the genus scale, earlier studies recovered corresponding CCDD cross-chromosome signals but differed in the inferred number and reciprocity of the underlying events [6,7]. Part of that disagreement may reflect reference dependence: without a directional reference, only the configuration relative to the genome chosen for comparison can be described, and the same segment may therefore be scored differently in different studies. The explicit ancestral polarity of the AOK integrates the previously reported signals into a single, more parsimonious model of three reciprocal translocations, and the distribution of the signals places that model on the ancestral branch shared by *O. alta* and *O. grandiglumis* after its separation from *O. latifolia*. At the population scale, the same directional reference is what allows segment orientation to be scored consistently across assemblies, because a call made relative to an extant genome depends on the arrangement that this reference carries.

### 3.2. Population-Scale Inversions: Lineage-Restricted and Functionally Heterogeneous

At the population scale, the four principal inversion-associated loci differed sharply in distribution, which is consistent with these variants being largely lineage-restricted rather than shared across the genus. The Chr2 and Chr9 loci were associated mainly with African rice, the Chr8 carriers were geographically clustered, and Chr6 stood apart because several nested or overlapping states occupied the same pericentromeric region. Previous inversion catalogs place this result in context: a pangenome inversion index built from 73 high-quality genomes identified 1769 non-redundant inversions spanning on average about 29% of the Nipponbare reference, implied an inversion rate of roughly 700 inversions per million years, 16–50 times higher than earlier estimates for plants, and showed effects on gene expression, recombination and linkage disequilibrium [21]. The four loci retained here are therefore a small, deliberately restricted subset of the structural variation present in the species: catalog-scale surveys quantify how much variation exists, whereas the AOK-polarized screen isolates the events whose polarity and cross-assembly support are strong enough to be interpreted individually.

The heterogeneity extended to gene content: functional annotation did not identify a single shared pathway, because the TE-related Pfam signal at Chr6 and Chr8 did not extend to a common GO or KEGG function. Because the screen retained only loci supported by several independent assemblies and the functional comparison rests on annotated protein-coding genes, these four loci are the principal well-supported events in this panel rather than a complete inventory of inversion-associated variation.

### 3.3. The Chr6 Hotspot: A Multiallelic Series and Its Phenotypic Context

At Chr6, AOK-polarized comparison organized previously reported configurations into one branching, multiallelic series [9,21,23,28] rather than a single binary inversion polymorphism. Using an ancestral reference helps to relate these separately reported configurations to one another, because each was originally described relative to a different reference. Inv13 marked African rice, whereas Inv15 and the Alt-Or topology were concentrated in *O. rufipogon*-related groups. On the Geng/*japonica* branch, Inv16 occurred with and without Big, but the Big interval occurred chiefly with Inv16; this asymmetry is most simply explained by placing Inv16 before Big in the structural path, although recurrent rearrangement or an unsampled intermediate cannot be excluded. The 5.24 Mb Big interval could suppress recombination and maintain linked genes or regulatory variants, as reported for comparable effects that contribute to supergene evolution in other systems [15,18,19]; recombination across the interval has not yet been measured, so this remains an expectation rather than an observation. Inv16-only and Big+Inv16 accessions had higher survival and lower injury scores than the predominantly non-GJ AOK-like group, and Big+Inv16 was also concentrated in GJ5 and temperate GJ. These phenotypic differences are associated with structural haplotypes, but their contribution cannot be separated from population background in this panel.

### 3.4. Limitations and Outlook

The AOK contains ordered genes rather than ancestral nucleotide sequence, so it cannot resolve noncoding evolution or the molecular composition of rearrangement breakpoints. The CCDD and Chr6 reconstructions suggest how the observed chromosome arrangements may have arisen from the ancestral state. Alternative histories remain possible, including recurrent rearrangement, convergence, lineage-specific loss and unsampled intermediates; wider taxon sampling and explicit transition models could distinguish among these histories. At the population scale, separating Chr6 structural effects from linked population variation will require population-aware association tests or segregating populations, and expression or phenotype comparisons among accessions with contrasting structural states under matched conditions would test whether the series has functional consequences. Integrating omics data with literature knowledge could further guide studies of stress-related traits [31]. Together with improved breakpoint-level resolution, these approaches could help move the present framework from ordering structural states towards testing their causes and effects.

## 4. Materials and Methods

### 4.1. Genome Data and Quality Assessment

Genome assemblies for AOK reconstruction were compiled from publicly released *Oryza* genome resources and accession- or DOI-linked repository records. The reconstruction panel comprised 24 chromosome-level genome assemblies. Allopolyploid assemblies were separated into their constituent subgenomes, yielding 34 ingroup genome/subgenome units for analysis. These units covered AA, BB, CC, BBCC, CCDD, EE, FF, GG, HHJJ and KKLL genome types. The telomere-to-telomere Nipponbare assembly was used as the NIP-T2T physical-coordinate reference. Unit-level source mapping is provided in Table S1. Project accessions, assembly accessions and DOI-linked records are listed in the Data Availability Statement.

The population-scale analysis combined two public resources: the 251-assembly rice super pangenome panel [10] and the 149-assembly wild and cultivated rice pangenome panel [11]. After sample-name harmonization, 400 unique sample identifiers defined the analysis universe. Reference genomes, duplicate records, cleaned-file aliases and records outside this manifest were excluded before state summarization. Frequencies were calculated with locus-specific callable denominators.

Completeness of the protein sets used for ancestral reconstruction was assessed with BUSCO v5.8.3 in protein mode using the embryophyta_odb10 lineage dataset (1614 markers) [32]; the two subgenome protein sets were combined for each allotetraploid genome, and the results are provided in Table S3. Contig N50, scaffold N50 and assembly gap counts were used as additional assembly-quality metrics.

### 4.2. Gene and Transposable Element Annotation

*Oryza* genome annotations from different pangenome projects were heterogeneous. Some wild-rice assemblies also lacked complete gene models. Where published annotations were available, we preferentially used those that were compatible with the corresponding genome assembly and had reported complete BUSCO scores above 90%; this criterion guided the selection of existing annotations and was not an exclusion threshold for the final gene sets. BUSCO scores for all 24 genome-level protein sets used here are given in Table S3. We therefore harmonized annotations and completed them where needed before orthology and synteny analyses. For assemblies with available protein-coding annotations, original GFF3, CDS and protein files were retained after gene-identifier normalization and longest-transcript selection. Where no suitable published annotation was available, gene models were generated from public RNA-seq data from the same species or the closest available *Oryza* material, as evidence for BRAKER3 v3.0.3-based gene prediction [33]. When species-matched transcriptome evidence was limited, closely related *Oryza* protein sets and high-confidence rice gene models were used as auxiliary homology evidence.

Four assemblies (*Oryza longiglumis*, *Oryza ridleyi*, *Oryza schlechteri* and *Oryza meridionalis*) lacked chromosome-compatible combinations of GFF3, coding-sequence and protein annotations, and no suitable transcriptome was available for them; their gene models were therefore generated by LiftOn v1.0.7-based annotation transfer [34]. The transferred models underwent the same formatting, identifier normalization and longest-transcript filtering. Genome sources, source accessions and species assignments are listed in Table S1. Final gene sets were converted to common GFF3, CDS and protein formats. The longest transcript of each gene was retained for OrthoFinder v3.1.4 [35], WGDI v0.75 [36] and downstream synteny analyses. This reduced bias from inconsistent transcript models across source projects.

Transposable elements (TEs) were annotated with a standardized workflow across all genomes. A non-redundant TE library was generated with EDTA v2.2.2 [37], and soft masking was performed with RepeatMasker v4.1.6 [38]. TE annotations were used for repeat masking before gene prediction and for characterization of breakpoint-proximal and inversion-interval repeat content.

### 4.3. Ancestral Genome Reconstruction

Ancestral genome reconstruction used the AGR workflow described by Siguret et al. [26], following the ancestral gene-order principles of Murat et al. [2]. Subgenomes of allopolyploid species were separated into independent analytical units, and each species or subgenome was treated as ploidy = 1 to avoid mixing chromosomes of different origins during reconstruction.

Orthogroups were inferred across the 34 ingroup genome/subgenome units derived from the 24 chromosome-level genome assemblies using OrthoFinder v3.1.4 [35]. Orthogroups present in at least 20 units were retained to build a binary chromosome-by-orthogroup matrix. AGR chromosome-correlation analysis and hierarchical clustering were then used to evaluate chromosome groups. The selected value, k = 12, was supported by a weighted silhouette score of at least 0.985. CAR extraction, fusion assessment, ancestor generation and synteny cleaning produced 12 contiguous ancestral regions and a 17,609-protogene pre-ancestor. Enrichment then added orthogroups represented in at least 18 units. This yielded the final AOK with 23,010 ordered protogenes on 12 protochromosomes. Final AOK inputs and ordered protogenes are provided in Table S1. *Oryza punctata* was represented by separate BB- and CC-derived analytical units.

### 4.4. Synteny Analysis and Rearrangement Identification

The AOK represents ordered protogenes, with coordinates defined by rank rather than nucleotide position. WGDI anchors derived from shared orthogroup membership were used only to project gene-order correspondence; their identity and score fields were placeholders, not measures of sequence similarity. WGDI v0.75 [36] projected extant chromosomes onto the ancestral chromosomes. Segment ancestry, order and orientation defined candidate rearrangements, which were recorded as AOK-polarized configurations rather than reconstructed ancestral haplotypes.

Candidate structural events were evaluated at both gene-order and nucleotide-sequence scales. Reversed segments from the same ancestral chromosome were classified as inversion candidates. Segments assigned mainly to a different protochromosome were classified as translocation candidates. WGDI blocks provided gene-level boundaries. MUMmer4 [39] and minimap2 v2.28 [40] alignments between NIP-T2T and representative assemblies assessed sequence orientation, breakpoint consistency and local assembly continuity. AOK-polarized comparison was used for polarity inference, whereas modern assemblies supplied physical coordinates and sequence-level evidence.

### 4.5. Population-Scale Structural Variation Analysis Across Rice Pangenome Assemblies

MUMmer4 alignments of the 400 assemblies to NIP-T2T were used to screen for large intrachromosomal reverse-orientation segments [39]. Sample names, chromosome labels, cleaned-file aliases and coordinate-column formats were harmonized between the two assembly panels. Alignments were generated with nucmer -c 30 -l 20, filtered with delta-filter -1 and exported with show-coords -THrd -r. Reverse blocks of at least 1 kb and at least 90% identity were grouped when separated by no more than 200 kb. Initial candidates required a span of at least 1 Mb, reverse coverage of at least 0.30, breakpoint clustering within 50 kb and support from at least three independent assemblies. Candidate loci were then evaluated by breakpoint compatibility, local alignment topology and AOK polarity.

Principal loci were re-audited with flanking sequence context. Final carrier assignment required a coherent reverse component, an event-compatible boundary and a supporting local topology. Chr6 was classified with breakpoint-family rules because this hotspot contains nested, partial and expanded configurations. These configurations cannot be represented by a single fixed-window call.

All 400 assemblies were retained in the population-scale screen. GJ1–GJ5 follow the 10,548-genome subgroup classification; independent 3K K9 assignments are reported separately in Table S2a. Six *japonica* assemblies from the 149-assembly panel lacked GJ1–GJ5 assignments and were classified as GJ-unassigned. The remaining eight accessions without supported population assignments were retained as unassigned. Five assemblies with insufficient or ambiguous local Chr6 alignment evidence were excluded only from the locus-specific denominator, leaving 395 callable assemblies, and were not counted as non-carriers. Low-frequency, combined and noncanonical configurations were checked with standardized local-reference and AOK-coordinate dot plots. Final sample-level structural-state calls are provided in Table S2.

Here, ‘recurrent’ refers to breakpoint-compatible configurations observed in multiple assemblies. Neighboring or overlapping sub-loci were collapsed using carrier-set similarity, coordinate overlap and gap criteria.

Compound and nested structures were evaluated separately from paired-breakpoint inversions. Five features were tracked at the Chr6 hotspot using projected NIP-T2T coordinates. Four were the Big interval (Inv6-Parent; 13.267–18.508 Mb), the Inv13 module (Inv6-Left; 13.569–13.756 Mb), the Inv15 module (Inv6-Central; 15.358–15.615 Mb) and the Inv16 module (Inv6-Right; 16.550–17.053 Mb). We also tracked the Alt-Or topology, a breakpoint-distinct alternative orientation spanning approximately 12.770–19.033 Mb. Hereafter, Big, Inv13, Inv15 and Inv16 denote these four features. Parent refers to the enclosing interval used for phase interpretation, not temporal priority. Local modules were assigned within the orientation context of the enclosing interval. Raw coordinate blocks and local dot plots defined Alt-Or as a distinct topology with two breakpoints outside the Big interval.

Breakpoint homology was assessed from local alignment topology and boundary coincidence. Canonical Inv13 comprised 17 full configurations, and two African accessions carried a shared partial subtype. Left-region configurations with distinct boundaries formed a separate complex class. Inv15 boundary differences of a few kilobases were grouped as subhaplotypes of the Inv15 module. Core and near-core Inv16 states formed one configuration class, whereas expanded Inv16-region topologies were recorded separately.

Chr6 structural states were assigned from the joint presence or absence of the Big interval, the Inv13 family, the canonical Inv15 module and Inv16-associated configurations. Recurrent four-feature combinations were designated structural haplotypes. The Alt-Or topology and other breakpoint-distinct, partial or complex configurations remained separate annotated classes rather than being forced into the binary feature scheme. Feature-based counts used the 395 callable assemblies. Alt-Or was summarized across all 400 assemblies because its distinct outer-breakpoint topology could be evaluated independently of complete feature callability.

Seedling cold-tolerance data were obtained from Shi et al. [30], who measured survival rate and leaf cold-injury score in 1992 accessions from the 3K Rice Genome Project. We matched the 251-genome panel to the published 3K records using source-panel cultivar names and the public BioSample-to-alias map; duplicated names were resolved only where the source-panel record supported a unique 3K alias. Of 170 matched accessions with callable states and non-missing phenotypes, 168 were AOK-like, Inv16-only or Big+Inv16 and represented the inferred Inv16-to-Big branch. Two Big-only accessions were retained in Table S2b but excluded because this class was too small for groupwise analysis. Each phenotype was compared across the three principal haplotypes using a Kruskal–Wallis test. These observational tests describe haplotype-associated distributions and do not estimate inversion-specific effects independently of population structure. Matched phenotype records are provided in Table S2b.

A rearranged region located approximately 2.1 Mb from the distal ends of the Chr11 and Chr12 short arms contains a long-range ectopic gene-conversion signal across *Oryza* species [4]. Structural classification in this region required two-ended breakpoints, sufficient reverse coverage and support across multiple assemblies.

### 4.6. Gene Content and Functional Enrichment of Inversion Intervals

Protein-coding genes were classified by NIP-T2T coordinates as internal, breakpoint-spanning or adjacent to each inversion interval. Breakpoint-spanning genes partially overlapped breakpoint coordinates, and breakpoint-proximal genes were defined as genes within ±100 kb of either breakpoint.

Functional annotations were unified with eggNOG-mapper v2.1.12 [41]. They included Pfam domains [42], Gene Ontology terms [43] and KEGG Orthology annotations [44]. Pfam-domain, GO-term and KEGG Orthology enrichment analyses were performed separately for each annotated locus; genes elsewhere on the same chromosome served as the comparison set. Fisher’s exact-test *p* values were adjusted with the Benjamini–Hochberg method [45], with a false discovery rate threshold of 0.05. Repetitive sequence features around breakpoints were analyzed by reciprocal BLAST+ (v2.2.31) comparisons [46] of ±100 kb sequences around the left and right breakpoints (word_size = 50). Direct and inverted repeats were summarized separately to identify sequence homology compatible with repeat-mediated rearrangement.

### 4.7. Statistical Analysis

Statistical analyses were performed in R v4.3.2 [47]. Statistical tests and multiple-testing procedures are specified with the corresponding analyses above.

### 4.8. Analysis Scale and Manual Curation

Principal loci were retained only when breakpoint compatibility, local alignment topology, assembly continuity, locus callability and cross-assembly support were all reviewable. Event rules and final sample-level state calls are provided in the Supplementary Materials.

## 5. Conclusions

The 12-chromosome AOK provides a shared polarity for structural comparisons across *Oryza* species and rice populations. It integrates the derived CCDD cross-chromosome signals into a parsimonious three-RCT model and resolves four principal inversion-associated loci among 400 assemblies. At Chr6, the observed states favor an Inv16-first path, and Big+Inv16 is concentrated in temperate Geng/*japonica* accessions with distinct published cold-response distributions. Segregating populations will be required to determine whether the structural haplotype contributes to these phenotypic differences independently of population background. More broadly, the AOK provides a framework for tracing chromosome rearrangements across evolutionary scales and testing how structural haplotypes contribute to rice diversification and trait variation.

## Data Availability

The public genome assemblies used for AOK reconstruction and their accession- or DOI-linked source records are listed in Table S1. NIP-T2T is available from NGDC (GWHBIPA00000000) and NCBI (GCA_034140825.1) [48]. Additional assemblies were obtained from Long et al. [6] (NCBI SRA PRJNA1175549; NGDC BioProject PRJCA024515; Figshare (https://figshare.com/s/32d0ac68cee7f647e1e3, accessed on 25 August 2026) and Fornasiero et al. [7] (PRJNA1016142, PRJNA687623, PRJNA439330 and PRJNA1115974). The population analysis used the 251-assembly rice super-pangenome panel [10] (PRJNA656318, PRJNA692836 and PRJCA004295) and the 149-assembly wild and cultivated rice pangenome panel [11] (PRJEB73710, PRJCA024131 and Figshare DOI: 10.25452/figshare.plus.25697817). The processed data generated in this study, including the complete 400-assembly AOK-coordinate dot plot overview (Data S1), are openly available on Zenodo at https://doi.org/10.5281/zenodo.20815410. The AOK protogene order, published population classifications, sample-level structural states and phenotype matches are provided in Tables S1 and S2; selected locus-level dot plots are provided as Data S2.

## Supplementary Materials

The following supporting information can be downloaded at https://www.mdpi.com/article/10.3390/plants15192989/s1, Figure S1: AOK-coordinate gene-order evidence for CCDD chromosomes; Figure S2: Representative AOK-coordinate Chr6 configurations; Table S1: Genome resources and AOK protogenes; Table S2: Sample structural states and cold-tolerance phenotypes; Table S3: BUSCO completeness of the 24 genome-level protein sets used for ancestral reconstruction; Supplementary Methods S1: Ancestral polarization, inversion discovery and structural-state classification; Data S2: Selected locus-level AOK-coordinate dot plots. The complete 400-assembly dot plot overview is archived as Data S1 on Zenodo (https://doi.org/10.5281/zenodo.20815410, accessed on 25 August 2026), as described in the Data Availability Statement.

## Abbreviations

The following abbreviations are used in this manuscript:

AGR	Ancestral Genome Reconstruction
AOK	Ancestral *Oryza* Karyotype
BLAST	Basic Local Alignment Search Tool
BUSCO	Benchmarking Universal Single-Copy Orthologs
CDS	Coding Sequence
EDTA	Extensive de novo TE Annotator
FDR	False Discovery Rate
GFF3	General Feature Format Version 3
GJ	Geng/*japonica*
GO	Gene Ontology
KEGG	Kyoto Encyclopedia of Genes and Genomes
KO	KEGG Orthology
LTR	Long Terminal Repeat
NIP-T2T	Telomere-to-Telomere Nipponbare Assembly
RCT	Reciprocal Chromosome Translocation
RNA-seq	RNA Sequencing
TE	Transposable Element
WGDI	Whole-Genome Duplication Integrated Analysis
