# Supplementary material for: Ancestral Karyotype Reconstruction Reveals Chromosome Rearrangements and Large Inversions in Oryza
Source: Plants (Basel). 2026 Sep 30;15(19):2989. doi: 10.3390/plants15192989 (PMC13666782; doi:10.3390/plants15192989)
Supplement: Supplementary file 1 [file plants-15-02989-s001.zip › Data_S2_selected_locus_dotplots.pdf]

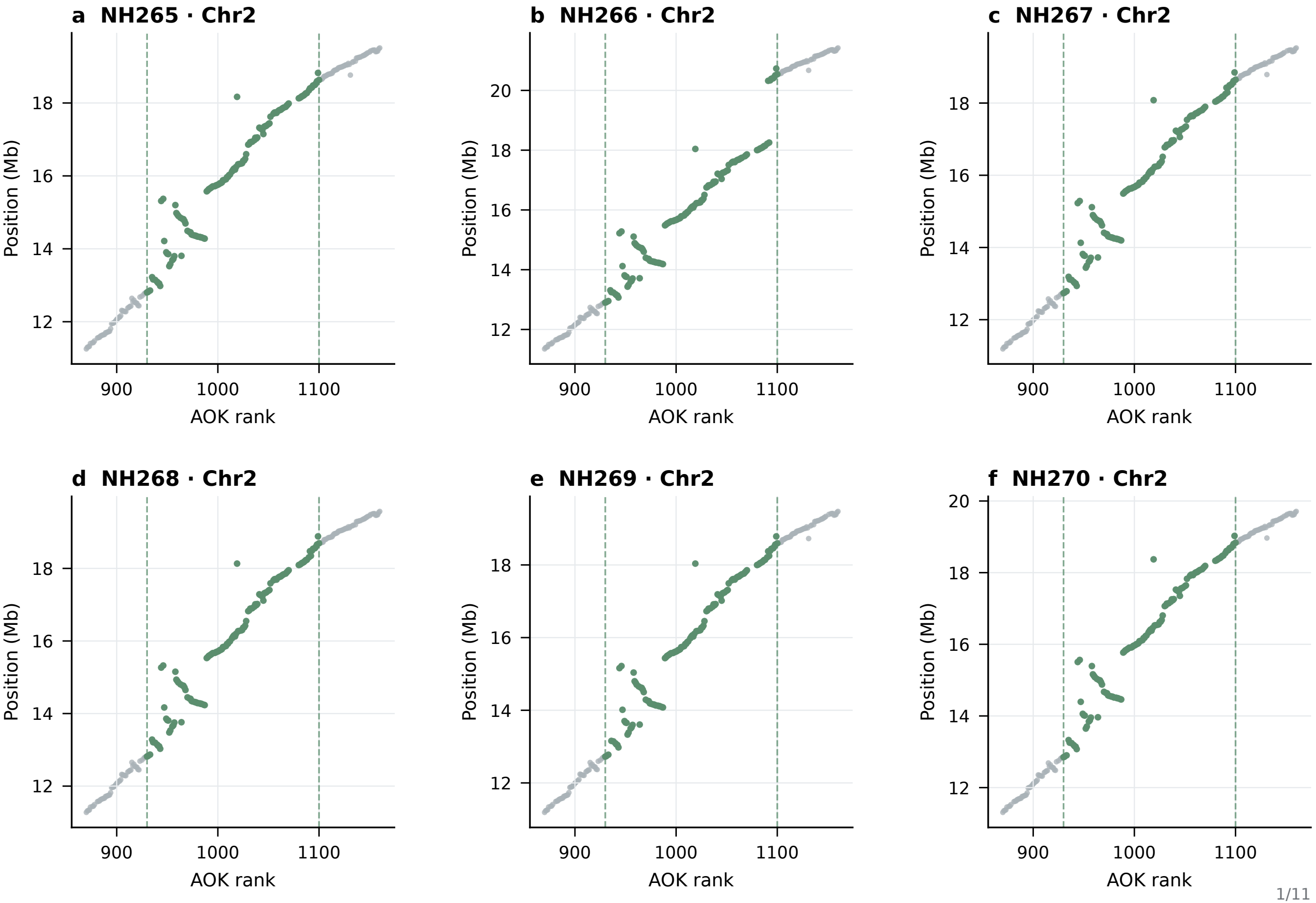

Data S2. Selected locus-level AOK-coordinate dotplots. Coloured points fall within the displayed event interval; grey points provide flanking context; dashed lines mark interval boundaries.

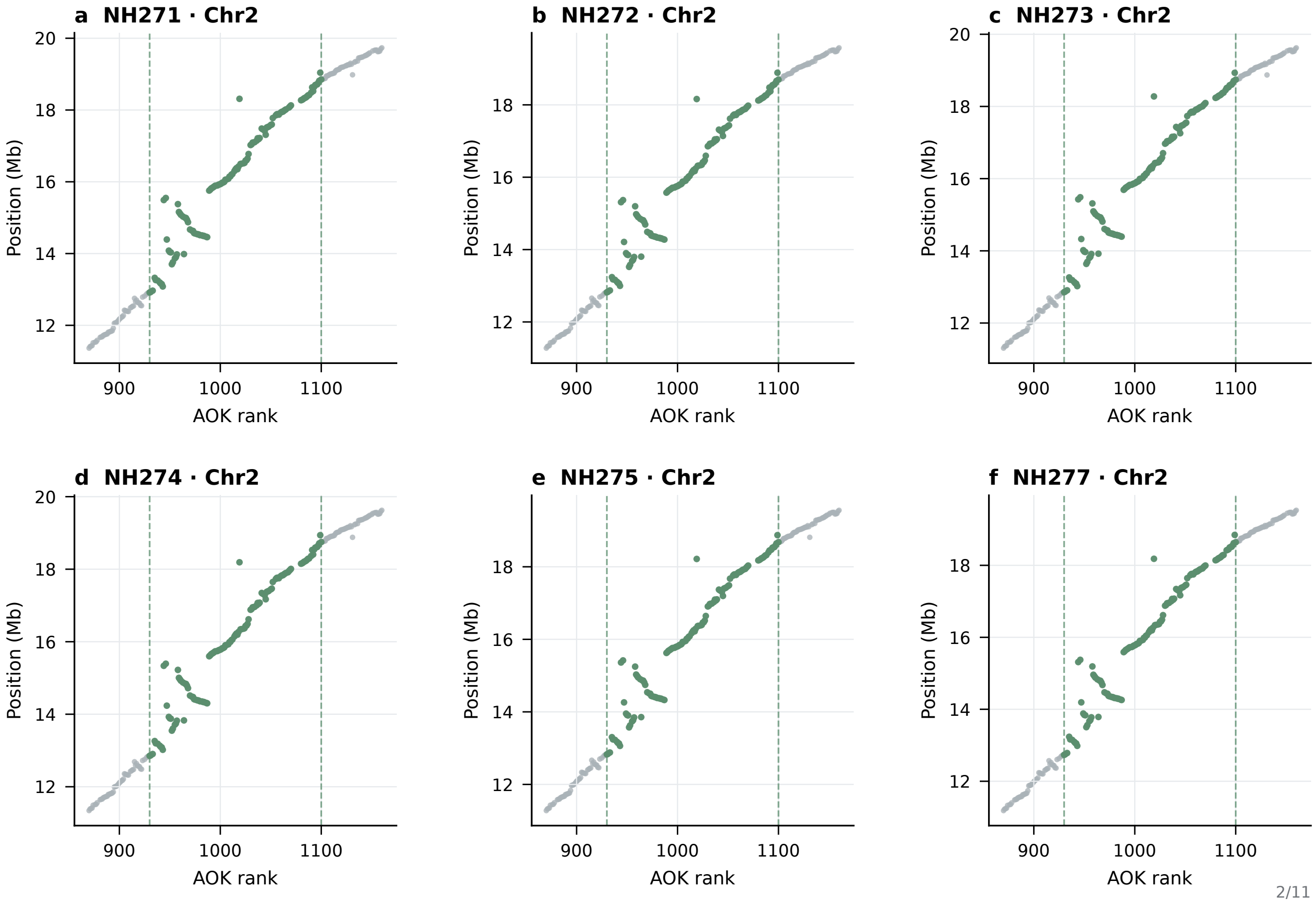

Data S2. Selected locus-level AOK-coordinate dotplots. Coloured points fall within the displayed event interval; grey points provide flanking context; dashed lines mark interval boundaries.

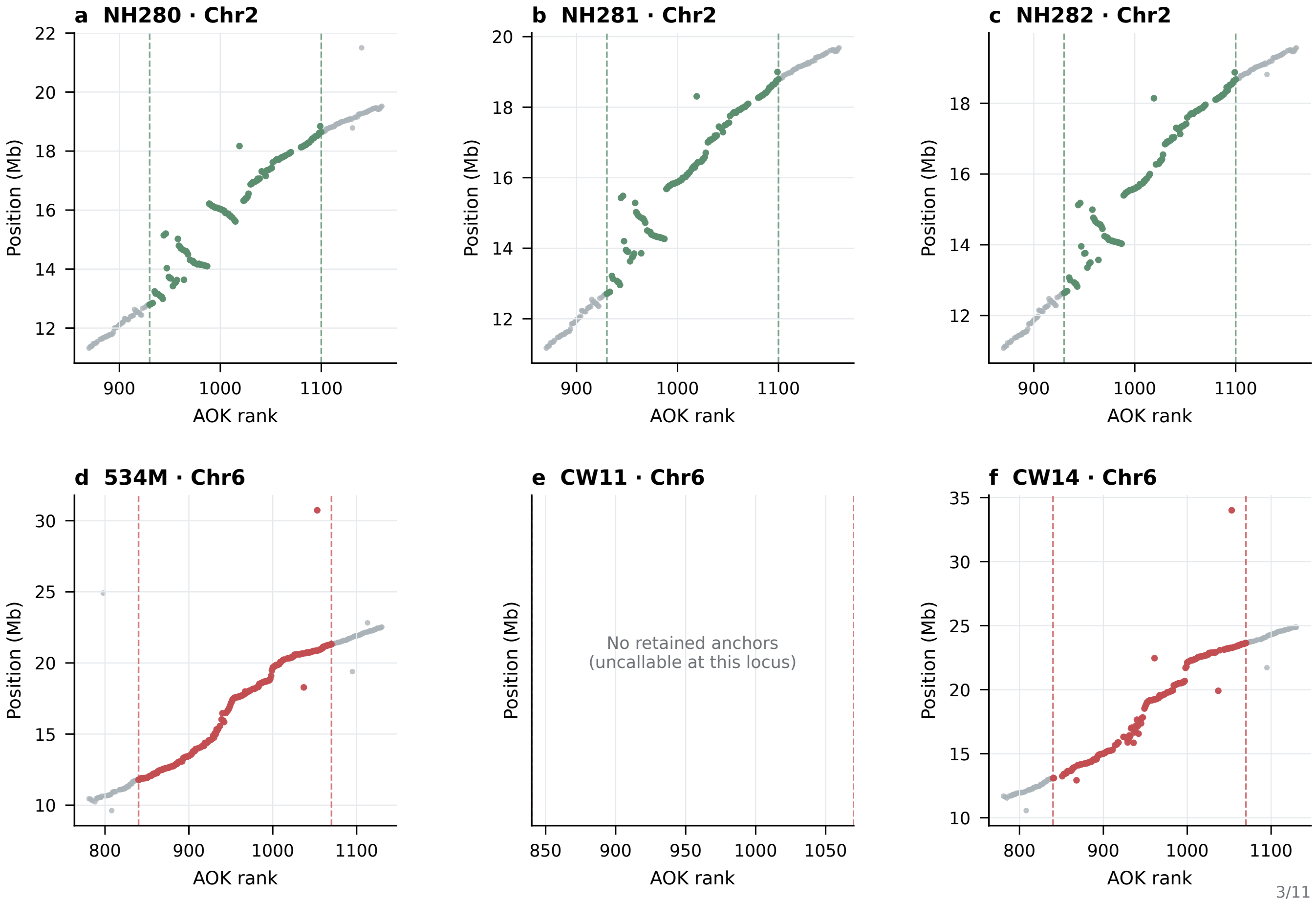

Data S2. Selected locus-level AOK-coordinate dotplots. Coloured points fall within the displayed event interval; grey points provide flanking context; dashed lines mark interval boundaries.

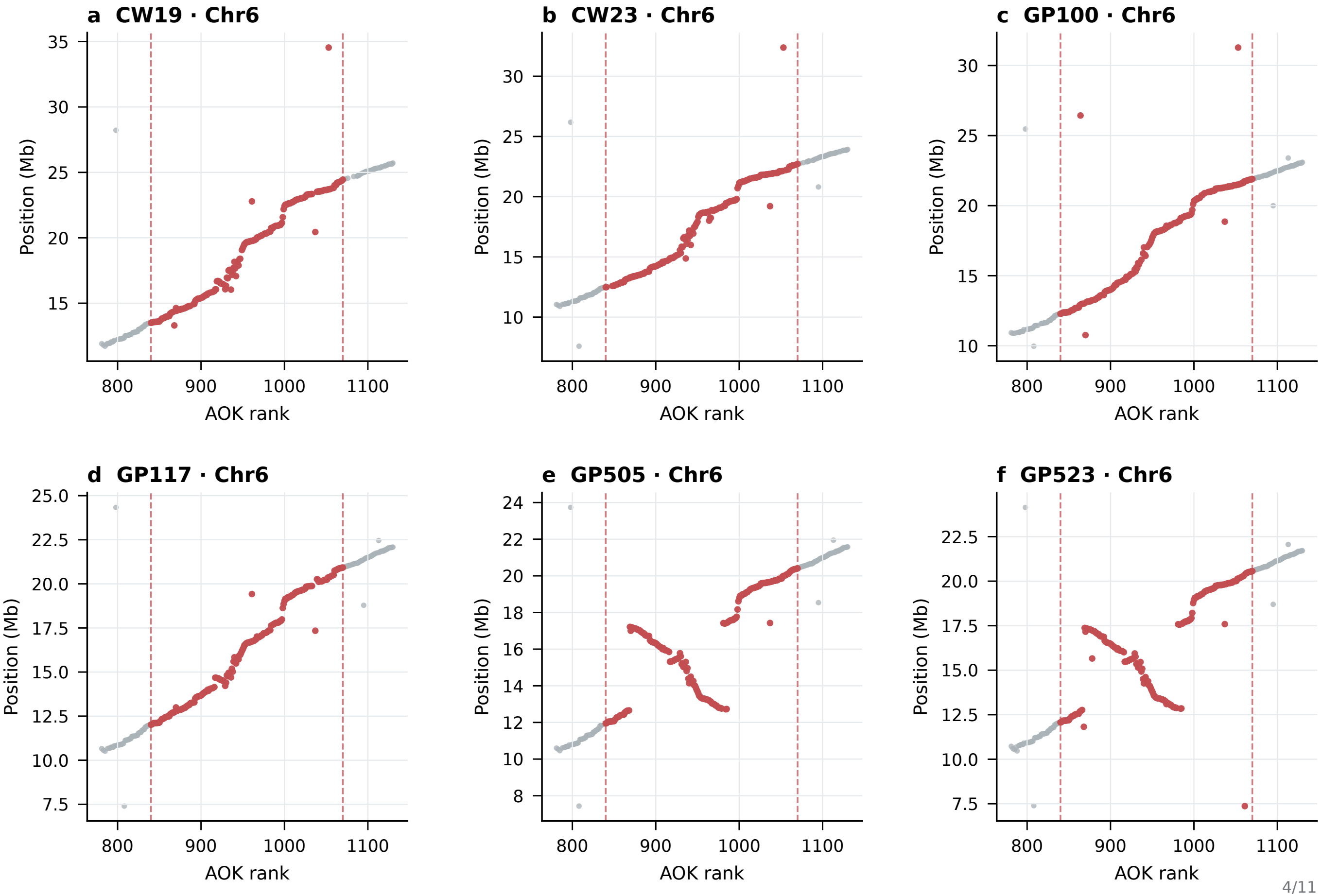

Data S2. Selected locus-level AOK-coordinate dotplots. Coloured points fall within the displayed event interval; grey points provide flanking context; dashed lines mark interval boundaries.

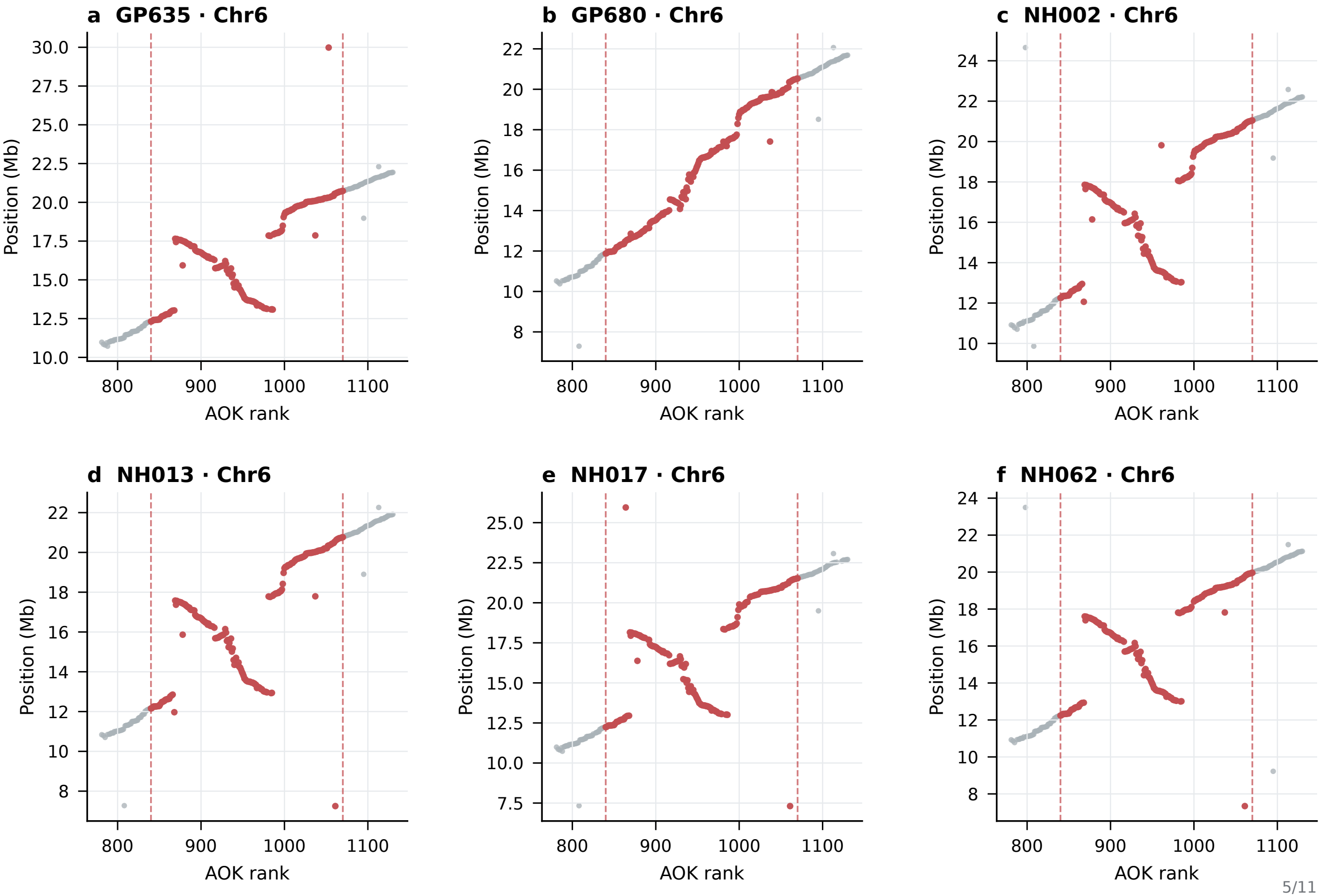

Data S2. Selected locus-level AOK-coordinate dotplots. Coloured points fall within the displayed event interval; grey points provide flanking context; dashed lines mark interval boundaries.

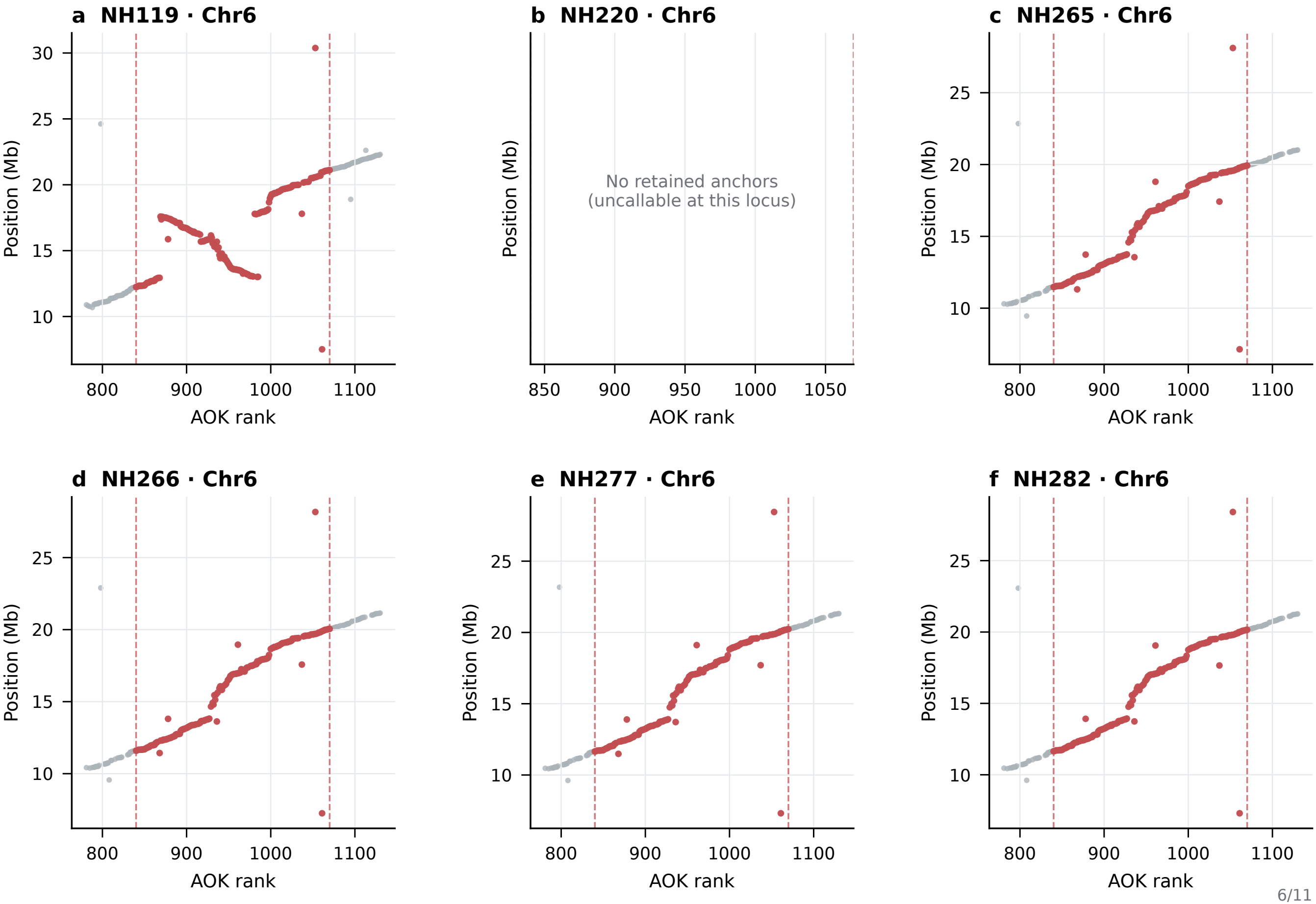

Data S2. Selected locus-level AOK-coordinate dotplots. Coloured points fall within the displayed event interval; grey points provide flanking context; dashed lines mark interval boundaries.

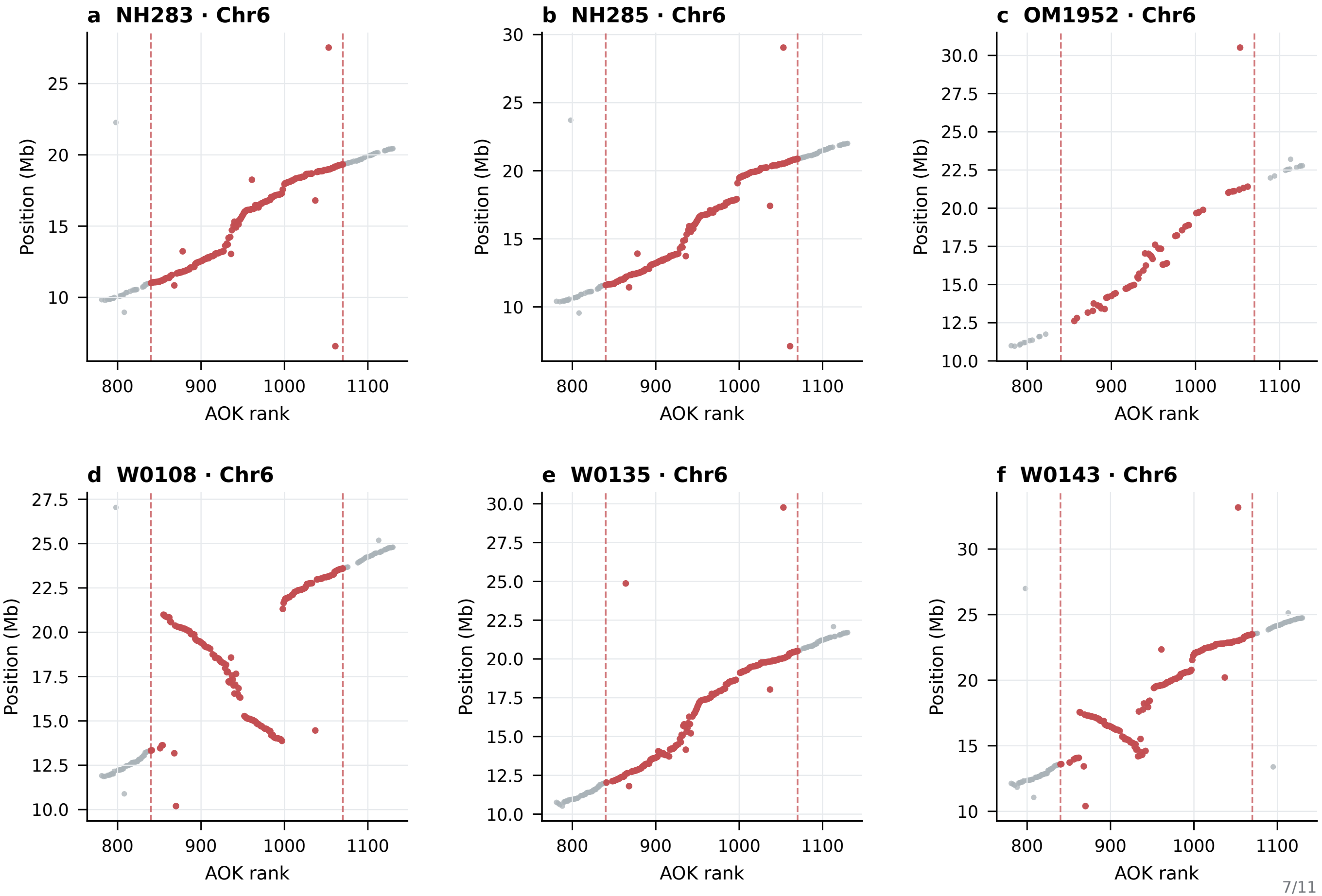

Data S2. Selected locus-level AOK-coordinate dotplots. Coloured points fall within the displayed event interval; grey points provide flanking context; dashed lines mark interval boundaries.

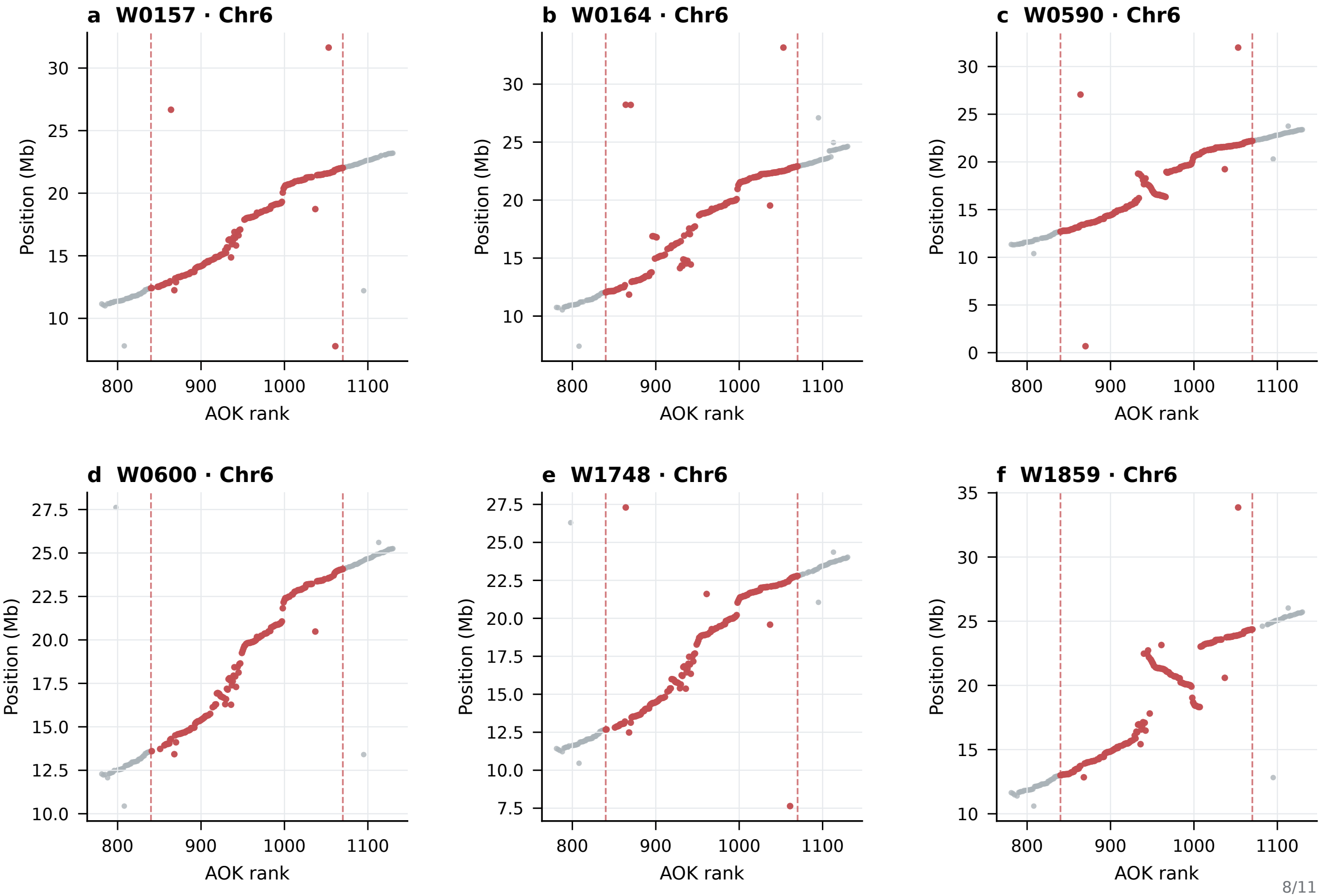

Data S2. Selected locus-level AOK-coordinate dotplots. Coloured points fall within the displayed event interval; grey points provide flanking context; dashed lines mark interval boundaries.

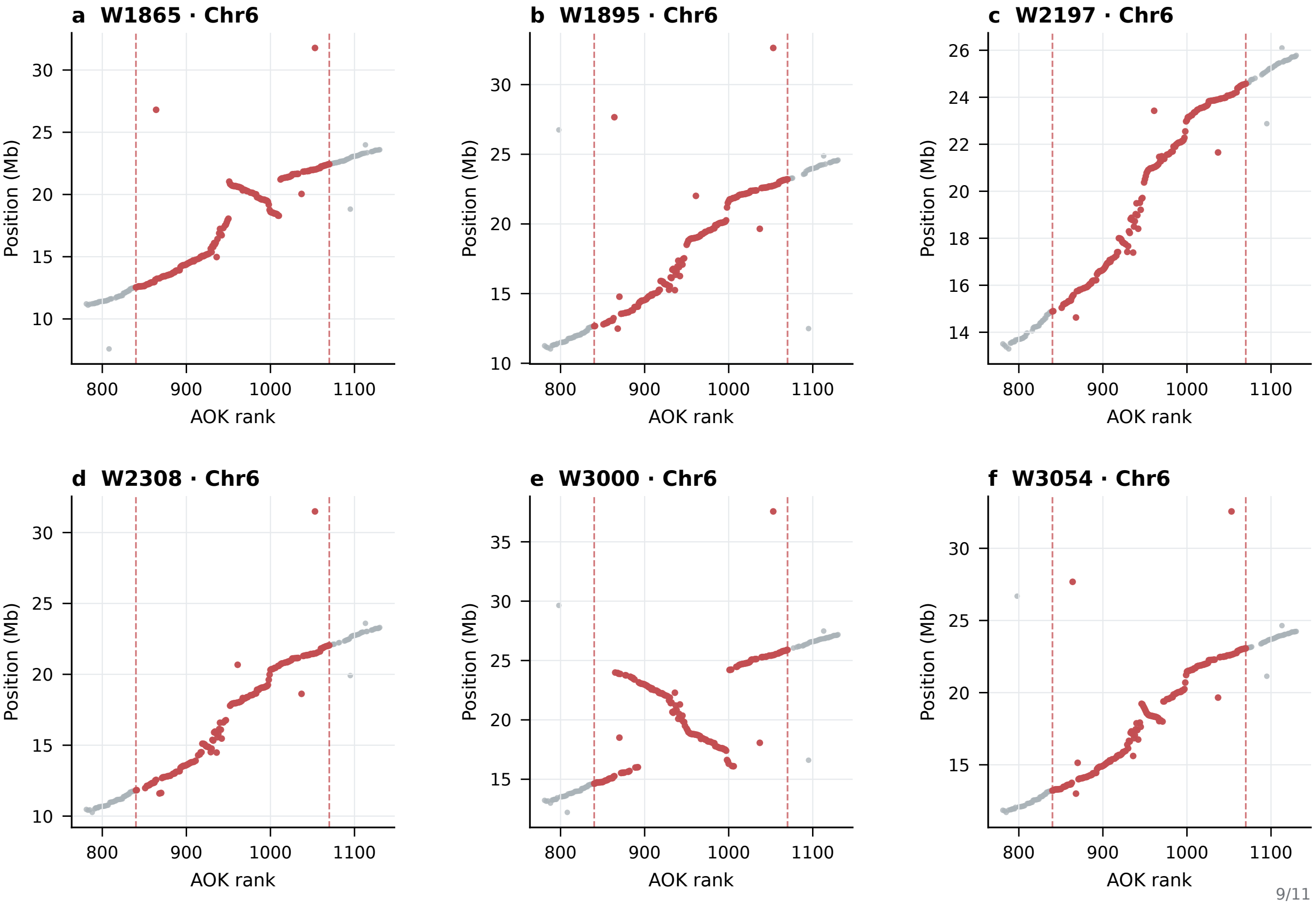

Data S2. Selected locus-level AOK-coordinate dotplots. Coloured points fall within the displayed event interval; grey points provide flanking context; dashed lines mark interval boundaries.

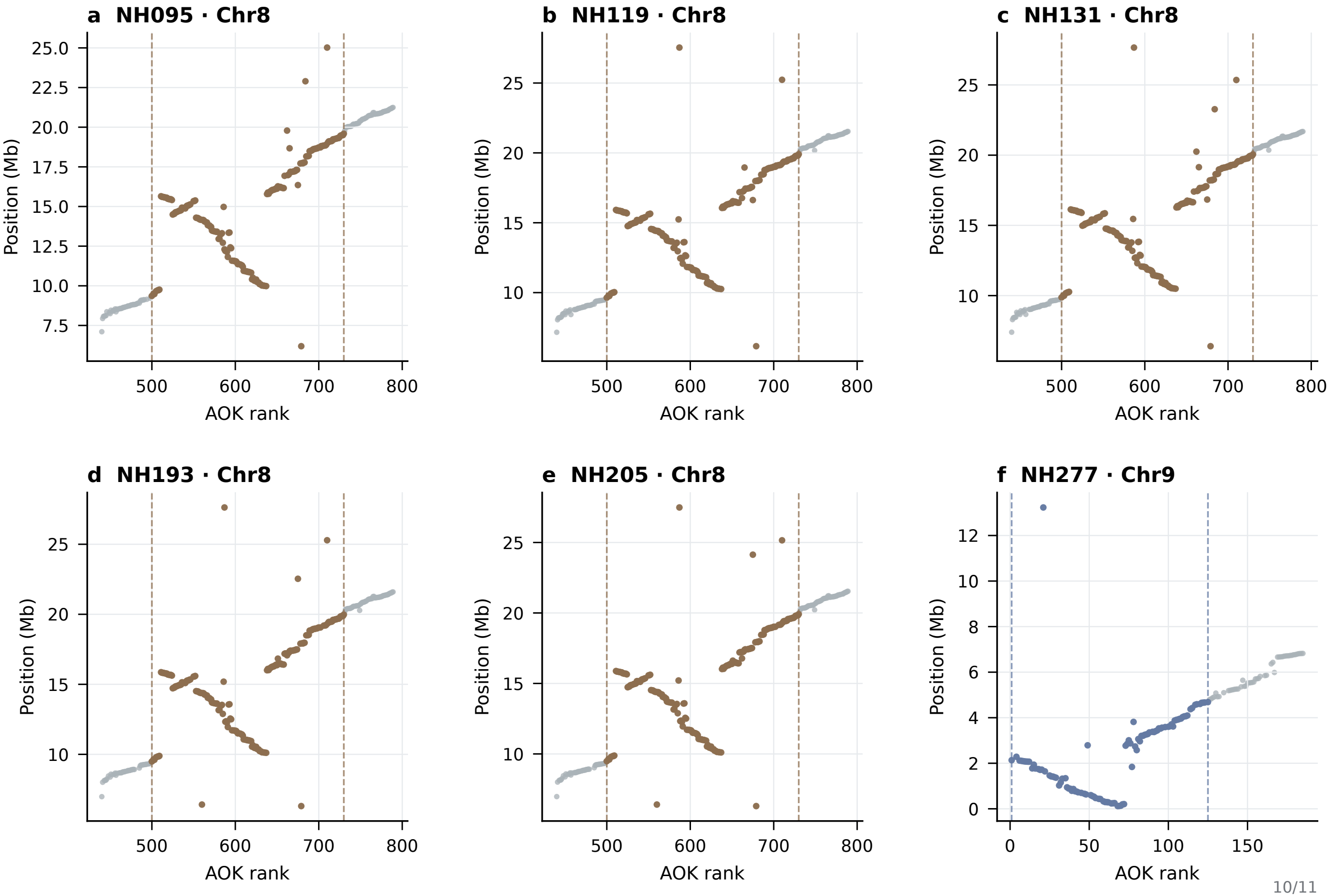

Data S2. Selected locus-level AOK-coordinate dotplots. Coloured points fall within the displayed event interval; grey points provide flanking context; dashed lines mark interval boundaries.

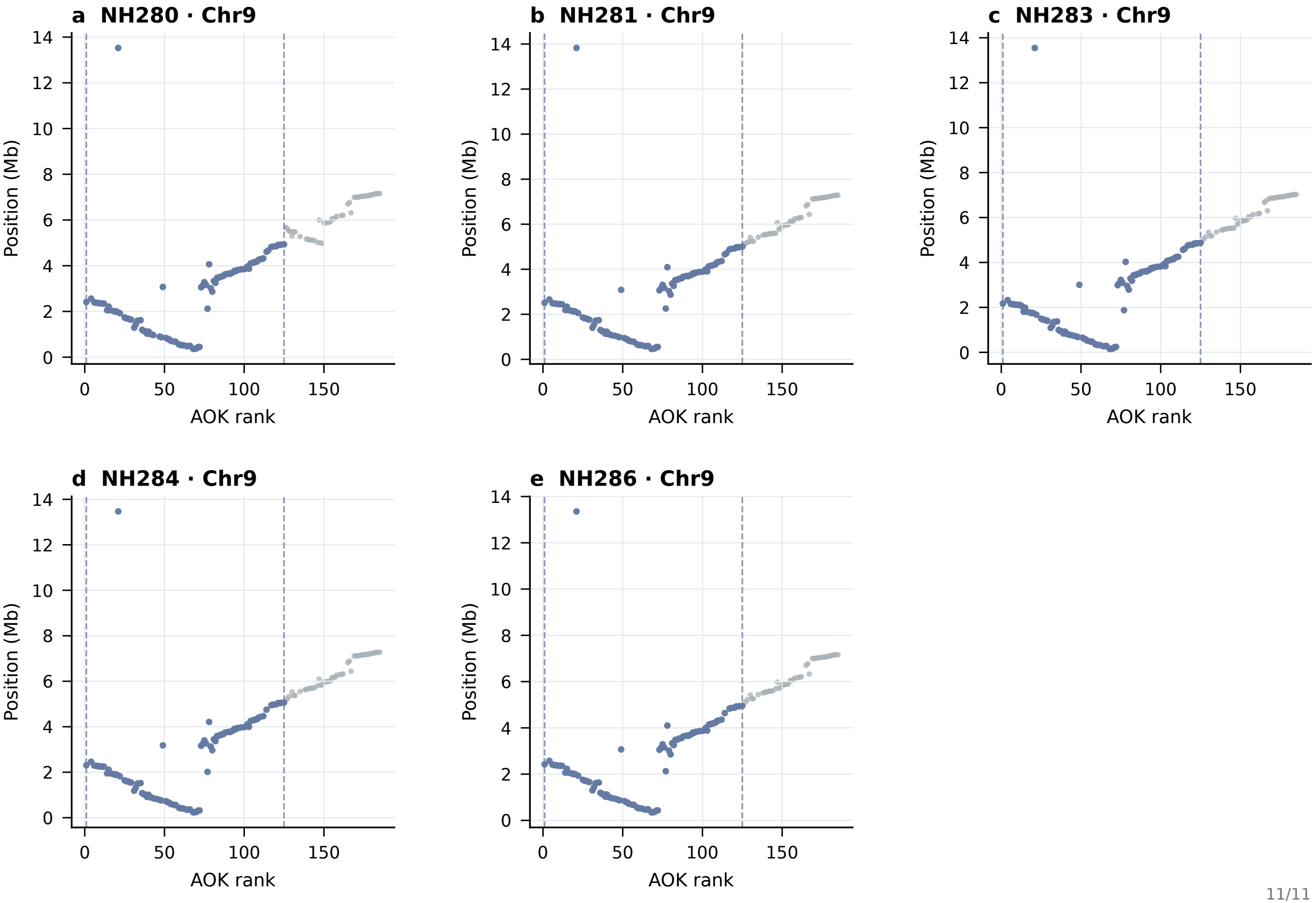

Data S2. Selected locus-level AOK-coordinate dotplots. Coloured points fall within the displayed event interval; grey points provide flanking context; dashed lines mark interval boundaries.
