# Supplementary material for: Ancestral Karyotype Reconstruction Reveals Chromosome Rearrangements and Large Inversions in Oryza
Source: Plants (Basel). 2026 Sep 30;15(19):2989. doi: 10.3390/plants15192989 (PMC13666782; doi:10.3390/plants15192989)
Supplement: Supplementary file 1 [file plants-15-02989-s001.zip › Figures_S1-S2.pdf]

Figure S1. AOK-coordinate gene-order evidence for CCDD chromosomes

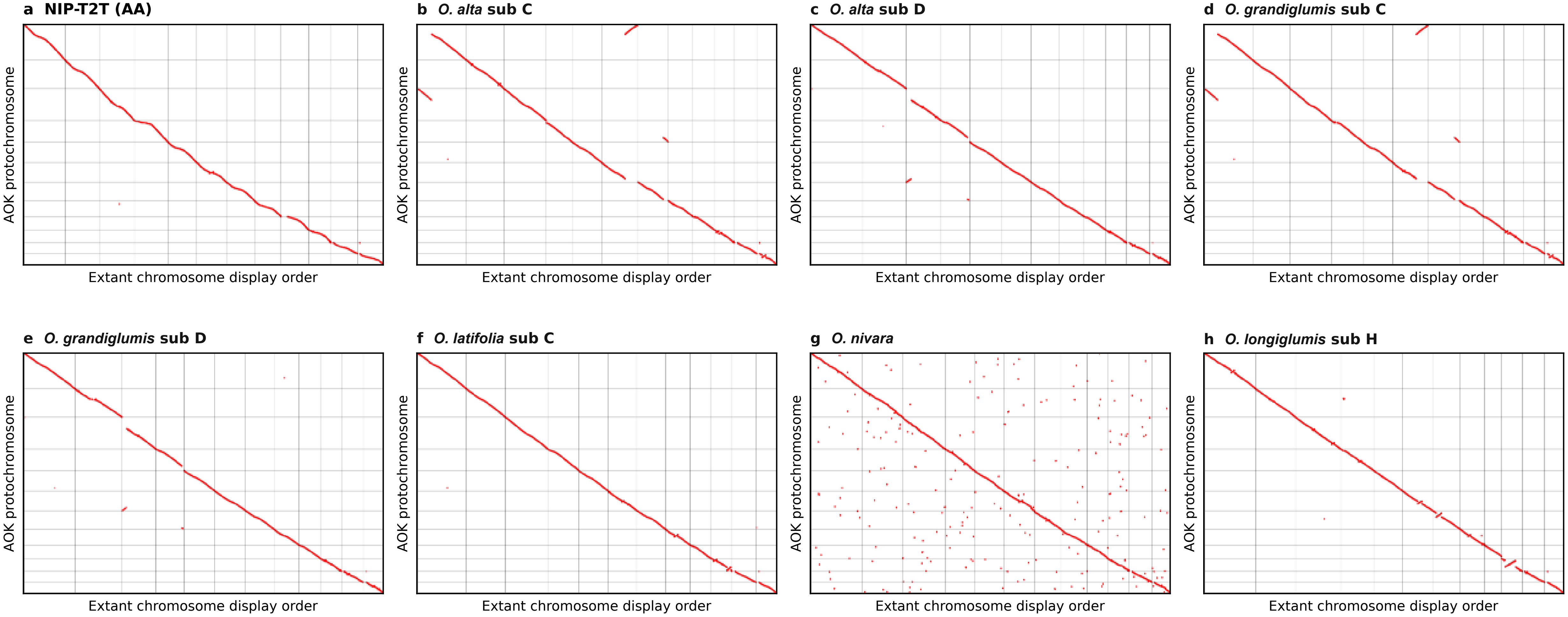

Panels show NIP-T2T, the C and D subgenomes of O. alta and O. grandiglumis, the C subgenome of O. latifolia, O. nivara and the H subgenome of O. longiglumis. The x axis follows extant chromosome display order and the y axis follows AOK protochromosome order. Anchors were derived from orthogroup membership; WGDI identity fields were artificial and were not interpreted as sequence similarity.

Figure S2. Representative AOK-coordinate Chr6 configurations

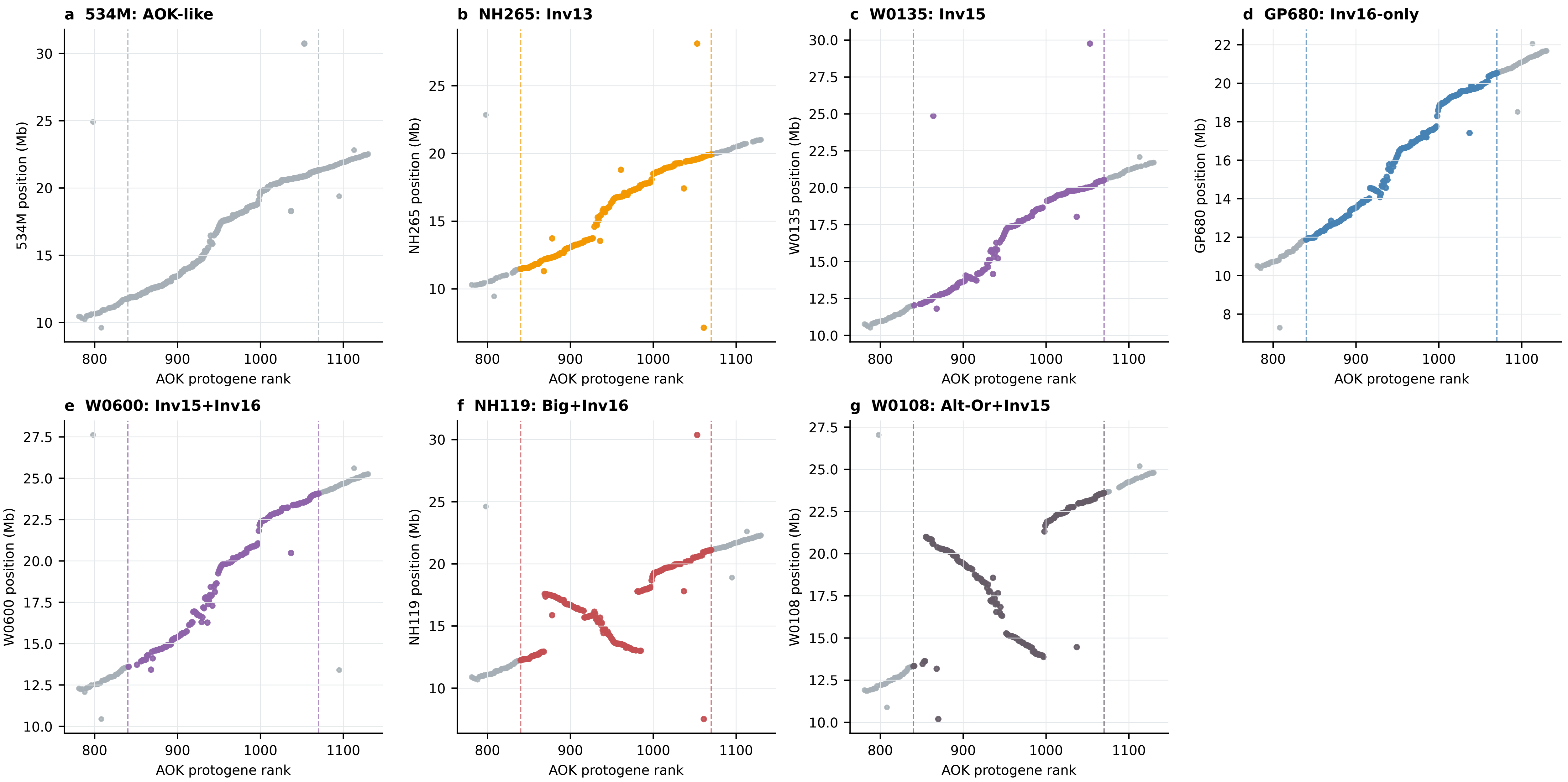

Panels show AOK-like, Inv13, Inv15, Inv16-only, Inv15+Inv16, Big+Inv16 and Alt-Or+Inv15 configurations. Colored points fall within the displayed event interval, whereas grey points provide flanking gene-order context. Dashed lines mark interval boundaries in AOK rank.
