# Supplementary material for: Ancestral Karyotype Reconstruction Reveals Chromosome Rearrangements and Large Inversions in Oryza
Source: Plants (Basel). 2026 Sep 30;15(19):2989. doi: 10.3390/plants15192989 (PMC13666782; doi:10.3390/plants15192989)
Supplement: Supplementary file 1 [file plants-15-02989-s001.zip › Supplementary_Methods_S1.pdf]

Supplementary Methods S1. Ancestral polarization, inversion discovery and structural-state classification

1. Scope and coordinate systems

The population analysis used an official manifest of 400 unique assemblies from the 251-assembly rice super-pangenome and the 149-assembly wild and cultivated rice pangenome. NIP-T2T coordinates were used to localize nucleotide alignments. AOK protogene rank was used to infer ancestral order and polarity and was not treated as a nucleotide assembly. Population-group labels and final sample-level calls are listed in Table S2 (sheet 'Sample structural states').

2. Whole-genome alignment and normalized reverse-block screen

Each assembly was aligned to NIP-T2T with MUMmer4 using nucmer -c 30 -l 20, delta-filter -1 and show-coords -THrd -r. Intrachromosomal reverse blocks were retained when they were at least 1 kb long and had at least 90% identity. Adjacent reverse blocks separated by no more than 200 kb were merged. Coordinate fields and sample names from the two source panels were normalized before screening.

3. Candidate thresholds, ancestral polarization and locus review

An initial candidate required a span of at least 1 Mb, reverse coverage of at least 0.30, breakpoint clustering within 50 kb and support from at least three independent assemblies. Candidates were projected onto AOK order through orthogroup-derived gene anchors. AOK-polarized comparison supplied polarity, whereas nucleotide alignments supplied physical boundaries and sequence orientation. Final calls required coherent reverse-orientation segments, event-compatible paired boundaries and a supporting local topology. The event rules are defined above, and final calls are listed in Table S2 (sheet 'Sample structural states').

4. Callability and denominator policy

All 400 assemblies remained in the analysis universe, with frequencies calculated from locus-specific callable denominators. A screening-level absence means that no configuration satisfying the relevant event rule was retained; it is not nucleotide-junction validation of a reference-like state. Five assemblies lacking sufficient or unambiguous Chr6 evidence were excluded only from the Chr6 feature denominator, leaving 395 callable assemblies, and were not counted as non-carriers. Alt-Or was evaluated in all 400 assemblies because its distinct outer breakpoints did not depend on complete four-feature callability.

5. Topology-aware classification of the Chr6 hotspot

Five structural features were tracked in projected NIP-T2T coordinates: the Big interval (13.267–18.508 Mb), three internal inversion modules—Inv13 (13.569–13.756 Mb), Inv15 (15.358–15.615 Mb) and Inv16 (16.550–17.053 Mb)—and the Alt-Or topology (approximately 12.770–19.033 Mb). Big, Inv13, Inv15 and Inv16 were classified by breakpoint-family compatibility and local topology. Alt-Or was retained as a separate broad topology because both outer breakpoints lay outside the Big interval. 'Parent' denotes an enclosing interval used for phase interpretation, not temporal priority. The Inv13 family comprised 17 complete states and two shared African-rice partial subtypes. Small Inv15 boundary offsets were grouped as subhaplotypes; core and near-core Inv16 states were combined, whereas extended or breakpoint-distinct topologies were annotated separately. Final per-assembly feature calls are provided in Table S2 (sheet 'Sample structural states').

6. Manual review and dotplot evidence

Low-frequency events, combinations, exceptions and ambiguous calls were reviewed with standardized local-reference and AOK-coordinate dotplots. Review considered paired-boundary coincidence, reverse-block continuity, forward alignment on both flanks, query-order disruption, local assembly continuity and agreement with AOK polarity. The complete 400-assembly AOK-coordinate overview (Data S1) is openly available on Zenodo (https://doi.org/10.5281/zenodo.20815410). Data S2 contains 65 selected sample-locus plots, including every Chr2, Chr8 and Chr9 carrier and rare, disputed, low-confidence or representative common Chr6 states.

7. Functional and breakpoint-context analyses

Protein-coding genes were classified as internal to an inversion-associated interval, overlapping a breakpoint or within ±100 kb of a breakpoint. Annotations were harmonized with eggNOG-mapper v2.1.12. Pfam-domain,

Gene Ontology and KEGG Orthology enrichment was performed separately for each annotated locus, using genes elsewhere on the same chromosome as the comparison set. Fisher's exact-test P values were adjusted by the Benjamini-Hochberg method at a false discovery rate of 0.05.

8. Published seedling cold-tolerance phenotypes

Seedling survival rate and leaf cold-injury score were obtained from Shi et al. (2024), who phenotyped 1,992 3K Rice Genome Project accessions. Records were matched to the 251-assembly panel using source-panel cultivar names and the public BioSample-to-alias map; duplicated names were resolved only where the source-panel record supported a unique 3K alias. The GJ1–GJ5 labels follow the independent 10,548-genome classification, whereas temperate (GJ-tmp), tropical (GJ-trp), subtropical (GJ-sbtrp) and admixed GJ (GJ-adm) follow the 3K K9 classification. Of 170 phenotype-matched, Chr6-callable accessions, 168 belonged to AOK-like, Inv16-only or Big+Inv16 and entered the three-group comparison. Two Big-only accessions remained in Table S2b but were excluded from groupwise analysis because the class was too small. Kruskal-Wallis tests were used. These tests describe haplotype-associated phenotype distributions and do not estimate an inversion effect independently of population structure.

9. Evidence map and interpretive boundary

Table S1 contains the 24 genome assemblies resolved into 34 ingroup genome/subgenome analytical units and the 23,010 ordered AOK protogenes; Table S2a contains the published 10,548-genome and 3K K9 classifications together with final sample-level structural states; Table S2b contains published cold-tolerance records used in Figure 4d. Figures S1 and S2 show CCDD gene-order evidence and representative Chr6 configurations, respectively. Data S1 is the complete 400-assembly overview archived on Zenodo, whereas Data S2 provides the selected locus-level dotplot atlas with the submission. The AOK is a gene-order reconstruction, not an ancestral nucleotide sequence. State histories are inferred from sampled topology, polarity and distribution; recurrent change, unsampled intermediates and lineage-specific loss remain possible. Table S3 reports BUSCO completeness of the 24 genome-level protein sets; subgenome protein sets were combined for each allotetraploid genome.
